# Genetic structuring and estimation of reproductive adults in *Onchocerca volvulus*: A genome-wide analysis across hosts and regions

**DOI:** 10.1371/journal.pntd.0013221

**Published:** 2025-07-01

**Authors:** Pawan Kumar, Young-Jun Choi, Kerstin Fischer, Shannon M. Hedtke, Anusha Kode, Nicholas Opoku, Lincoln Gankpala, Tony O. Ukety, Jöel Lonema Mande, Timothy J.C. Anderson, Warwick N. Grant, Peter U. Fischer, Makedonka Mitreva

**Affiliations:** 1 Division of Infectious Diseases, Department of Medicine, Washington University School of Medicine, St. Louis, Missouri, United States of America; 2 Department of Evolution and Genetics, School of Agriculture, Biomedicine and Environment, La Trobe University, Bundoora, Victoria, Australia; 3 Fred Newton Binka School of Public Health, University of Health and Allied Sciences, Ho, Ghana; 4 Division of Public Health and Medical Research, National Public Health Institute of Liberia, Charlesville, Liberia; 5 Centre de Recherche en Maladies Tropicales, Rethy, The Democratic Republic of Congo; 6 Program in Disease Intervention and Prevention, Texas Biomedical Research Institute, San Antonio, Texas, United States of America; 7 McDonnell Genome Institute, Washington University in St. Louis, St. Louis, Missouri, United States of America; Instituto Leonidas e Maria Deane / Fundacao Oswaldo Cruz, BRAZIL

## Abstract

Genomic analysis of parasites can deepen our understanding of their transmission, population structure, and important biological characteristics. Onchocerciasis (river blindness), caused by the parasitic nematode *Onchocerca volvulus*, involves adult worms residing in subcutaneous nodules that produce larval-stage microfilariae (mf), which are routinely detected in the skin for diagnosis. Whole-genome studies of mf are limited; most analyses have focused on the mitochondrial genome. We conducted a genome-wide analysis with 94% median nuclear genome coverage, analyzing 171, 37, and 98 mf from 16, 3, and 5 individuals from Ghana, Liberia, and the Democratic Republic of Congo, respectively. These data were used to investigate population differentiation, estimate the number of reproductive adult worms, and analyze genetic variation across chromosomes. Population genetic analyses across hosts and countries showed that nuclear genome diversity can reveal fine-scale genetic structure, even between geographically close countries, providing more resolution than mitochondrial haplotype data. By reconstructing maternal and paternal sibships, we estimated the number of reproductively active adult filariae. Comparisons between adult worm estimates from genetic data and nodule observations showed that genetics-based estimates were higher or equal to observed worm counts in 8 out of 9 hosts for female worms and 7 out of 9 hosts for male worms. Our analysis also revealed lower-than-expected X chromosome diversity, consistent with neo-X chromosome fusions in filarial species. This study represents an important step in using nuclear genome data from mf to support onchocerciasis elimination efforts and in developing genetic tools that could inform mass drug administration programs.

## Author summary

*Onchocerca volvulus*, a human filarial parasite, causes river blindness, a neglected tropical disease that primarily affects sub-Saharan Africa. Despite extensive efforts to eliminate the disease through mass administration of ivermectin—which interrupts parasite transmission by blackfly vectors by killing and reducing the production of larval parasites (microfilariae) but does not eliminate adult worms—some regions continue to experience persistent transmission or resurgence. Genetic data can provide valuable epidemiological insights that are otherwise challenging to obtain, such as estimates of adult worm burden and fecundity in individual hosts. By analyzing microfilariae samples collected from clinical trials, we demonstrate that it is possible to genetically estimate the number of reproductively active adult worms from these samples. Our whole nuclear genome analysis of microfilariae also highlights the benefits of high-resolution analysis of genetic diversity and differentiation, surpassing the limitations of previous studies. These findings represent a crucial first step in developing genetic tools for analyzing longitudinal microfilariae samples to assess treatment effects and identify new infections, ultimately aiding in the monitoring and improvement of mass drug administration programs.

## Introduction

Onchocerciasis, or river blindness, is caused by the filarial nematode parasite *Onchocerca volvulus*. Infection may cause pathologies including skin de- or hyper-pigmentation, severe dermatitis, pruritus, epilepsy, Nakalanga syndrome, irreversible blindness, and social ostracization [1,2]. It is a neglected tropical disease (NTD) and the second-leading cause of irreversible blindness worldwide. In 2022, the World Health Organization (WHO) estimated that at least 246 million people lived in communities that should be targeted for mass drug administration with ivermectin (MDAi) [3]. Ivermectin kills most of the first stage larvae (microfilariae, mf) in the skin and is embryostatic, acting as a temporary contraceptive for female worms. MDAi thus interrupts the transmission cycle because there are no mf for the blackfly vectors to transmit [4–6], and is therefore the main strategy used in efforts to eliminate onchocerciasis [7,8]. To date, four countries in the Americas (Colombia, Ecuador, Guatemala, and Mexico) have been WHO-verified for having successfully eliminated transmission of onchocerciasis [3,9]. In sub-Saharan Africa, where >99% of people who are at risk of onchocerciasis reside, over 160 million people were treated in 2022, 16 countries achieved 100% geographical coverage, and Niger has submitted a dossier for verification of elimination [3].

Rapid Epidemiological Mapping of Onchocerciasis (REMO) was initially used to identify meso- and hyperendemic *O. volvulus* foci that were prioritized for intervention [10]. Adult female *O. volvulus* form subcutaneous nodules, known as onchocercomas, that can be readily palpated. Nodules that are not externally visible or palpable are sometimes located in deeper tissues and are often attached to bones [11–13]. Male worms migrate between nodules, where reproductively active, sessile females reside [14]. Fertilized female worms release thousands of mf into the skin [11], which then disperse throughout the body (although the rate of dispersal is not known). Skin snips are often taken from four different parts of the body (e.g., the iliac crest and calf) to assess the presence of skin mf. The number of mf in a skin snip can be counted as a proxy for worm burden; however, such counts do not account for variability in adult reproductive output or in immune response to mf, and may not accurately reflect the true adult worm burden.

In Africa, there remain areas where transmission persists despite MDAi for longer than the ~ 15-year lifespan of the adult worms, or where transmission has resumed after MDAi has been stopped [6,15–21]. Furthermore, so far little or no MDAi has been performed in areas with hypoendemic onchocerciasis or in areas coendemic for loiasis where there is an elevated risk of serious adverse events after administration of ivermectin. Ongoing transmission can be associated with the operational challenges involved in running decades-long MDAi programs, particularly in areas with high onchocerciasis endemicity requiring high coverage and many repeated rounds of ivermectin. In addition, sub-optimal responses of adult female worms to the contraceptive effect of ivermectin and sub-optimal mf killing of ivermectin have been reported [22–31]. Transmission can also be re-introduced to a focus after MDAi has been stopped due to either migration of infective vectors of *O. volvulus* (blackflies in the genus *Simulium*) from a neighboring focus in which transmission continues, or when infected people move between communities (often for farming work) [16,32,33].

Genetic epidemiology can be applied to investigate ongoing transmission despite MDAi and post-MDAi recrudescence [34]. Through longitudinal sampling of mf, genomic approaches can distinguish between adult worms that continue to reproduce after multiple rounds of therapy and new infections resulting from ongoing transmission, while also tracking the decline in female reproduction over time—a critical challenge in evaluating the impact of MDAi [35]. This strategy would enable direct monitoring of drug efficacy on female fertility during clinical trials and MDAi, a capability that is currently unavailable. Population genetic data has been used to explore how parasites have moved throughout the landscape in West Africa [24,36,37]. In Ghana, for example, similarities in genetic variation between river basins in a central transect spanning the ecological transition zone between savannah and forest ecosystems suggested that parasites have been moving via infected people or infective vectors throughout a 250-km transect [24,37,38]. However, worms sampled from Ghana are genetically distinct from worms from Mali, Cote d’Ivoire, Cameroon, and the Democratic Republic of the Congo (DRC) [24,37,39], suggesting that people and vectors are not regularly moving between Ghana and other countries. Population genetic data has also been used to estimate the number of reproductively active female worms in a person and has shown that the worm burden is higher in South Sudan than in the DRC [39]. Tracking the number of reproductively active female worms may be more informative about the success of ongoing MDAi than counts of the mf burden in the skin [39].

Previous population genetic research, however, has focused on either very small sample sizes within a country [36], pooled genome coverage with low depth per worm [24], or exclusively on mitochondrial data [37,39]. Mitochondrial data are limited, as they can only track maternal lineages (mitochondria are maternally inherited in *O. volvulus*), are inherited as a single locus, and have far fewer variable sites than the much larger nuclear genome inherited from both parents. Here, we demonstrate the increased resolution possible using nuclear genome data from mf to evaluate genetic variation and population structure in *O. volvulus*. The goals of this study were to i) investigate the nuclear and mitochondrial genetic differentiation among mf within hosts, between hosts within a country, and between countries, ii) estimate the minimum number of reproductively active adult female and male worms in a host based on mf genotypes and iii) examine the genetic diversity and patterns of LD in the autosomes and X-chromosome in *O. volvulus* using high-coverage diploid genome data. These analyses were facilitated by whole-genome sequencing of mf sampled from participants in historical samples or clinical trials in West Africa (Liberia and Ghana) and clinical trials in Central Africa (eastern DRC).

## Results

### Whole-genome amplification and sequencing of individual microfilaria

**Fig 1:**
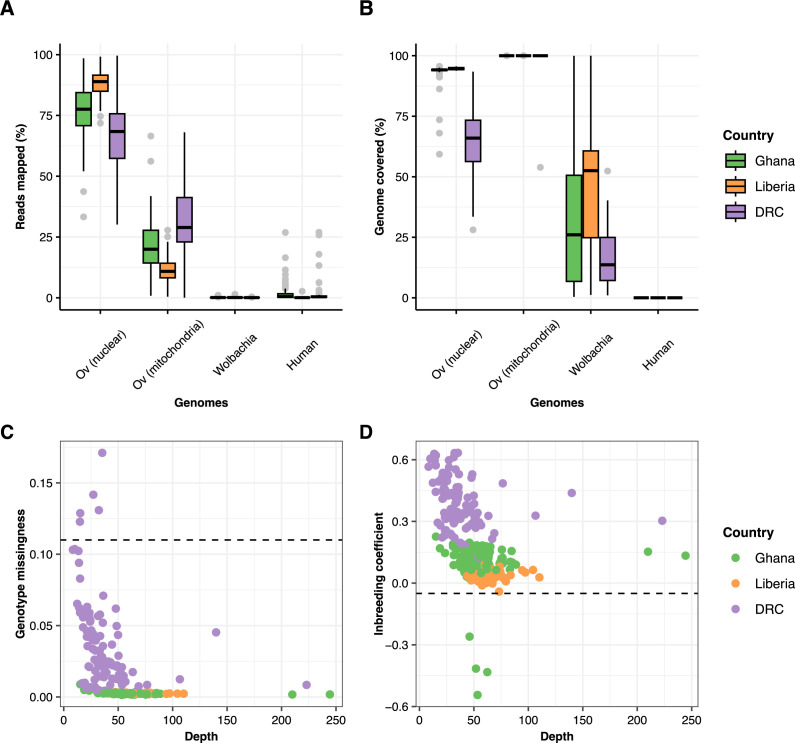
Library composition and genome coverage of *Onchocerca volvulus* microfilariae samples (n = 315), and the criteria for sample exclusion. (A) Proportion of reads mapped to *O. volvulus* (Ov) nuclear and mitochondrial genomes, endosymbiont *Wolbachia*, and *Homo sapiens*. (B) Breadth of coverage for each of the four genomes at a minimum depth of 10X. (C) Exclusion of samples with a high rate of missing genotype calls (>11%). (D) Exclusion of samples with highly negative inbreeding coefficient values (<-0.05), indicative of possible sample contamination. Cutoffs for exclusion (panels C and D) are represented by dashed lines.

We sequenced the amplified genomes of 315 mf isolated from 24 participants from 3 countries, Ghana (n = 16), Liberia (n = 3) and the DRC (n = 5) (Tables 1 and S2). The median percentages of reads mapped to the nuclear, mitochondrial, and *Wolbachia* genomes of *O. volvulus* were 76.3%, 22.4%, and 0.11%, respectively (Fig 1A). The median breadth of genome coverage with a depth greater than 10X was 94%, 100%, and 23.3% for the nuclear, mitochondrial, and *Wolbachia* genome of *O. volvulus*, respectively (Fig 1B). We did not observe a significant bias in the distribution of male and female mf in any of the participants (binomial test, *P* ≥ 0.05 when compared to the expected 50:50 male:female ratio) (S1 Fig). No significant bias was observed across all hosts (*P* ≥ 0.05). Of the 315 sequenced mf, 9 samples were excluded due to high rates of missing genotype calls (>11%) or substantially negative inbreeding coefficients, which indicated potential mixing of distinct samples (Fig 1C and 1D), resulting in 306 samples (Ghana = 171, Liberia = 37, and DRC = 98) from 24 participants to be retained for further analysis (Table 1).

**Table 1:** Number of *Onchocerca volvulus* microfilariae (mf) samples sequenced and analyzed from participants from three African countries.

Country	Number of participants	Participant ID	Number of mf sequenced		Number of mf analyzed	
Ghana	16	GH_1010	3	175	3	171
		GH_1013	7		7	
		GH_1015	7		7	
		GH_1036	1		1	
		GH_1086	6		6	
		GH_1118	30		29	
		GH_1123	12		12	
		GH_1161	12		12	
		GH_1171	10		10	
		GH_1174	12		12	
		GH_1177	22		22	
		GH_1182	2		2	
		GH_1213	7		7	
		GH_1219	10		9	
		GH_1224	21		19	
		GH_1250	13		13	
Liberia	3	LR_320472	14	37	14	37
		LR_320562	4		4	
		LR_320573	19		19	
DRC	5	DRC_1118	55	103	53	98
		DRC_1123	38		35	
		DRC_1157	1		1	
		DRC_1231	6		6	
		DRC_1284	3		3	
Total	24			315		306

### Mitochondrial and nuclear genetic diversity in *O. volvulus* across geographic regions

For mitochondrial data, we excluded 137 singleton SNPs and one mf sample from participant DRC_1231 (due to >5% missing variants sites); 305 mf and 206 variant sites in the 13,744 bp mitochondrial genome remained. For nuclear data, 3.4 million SNPs (2.6 million autosomal, 0.75 million X-linked, and 0.03 million Y-linked variants) were called. We excluded nine low-quality mf samples (Fig 1C, 1D, and S2 Table), the sex chromosomes, and sites in linkage disequilibrium; 9227 autosomal variants (minor allele frequency >5%) and 306 individual mf remained (Table 1). Since mitochondrial data are haploid and does not require phasing, we were able to perform haplotype-based analyses. Samples from the DRC formed a clearly differentiated, separate cluster in the network, while haplotypes from Ghana and Liberia clustered together (S2A Fig). We observed 44, 12, and 28 unique haplotypes within each country, with nucleotide diversities (π; the average number of pairwise nucleotide differences per site) of 0.034, 0.030, and 0.044 for Ghana, Liberia, and the DRC, respectively. There were 5, 1, and 1 unique haplotypes shared by multiple hosts in Ghana, Liberia, and the DRC, respectively (S2B–S2D Fig). These results suggested that mitochondrial haplotypes could be used to differentiate West African *O. volvulus* populations in Ghana and Liberia from the DRC population in Central Africa, but not between Ghana and Liberia populations.

**Fig 2:**
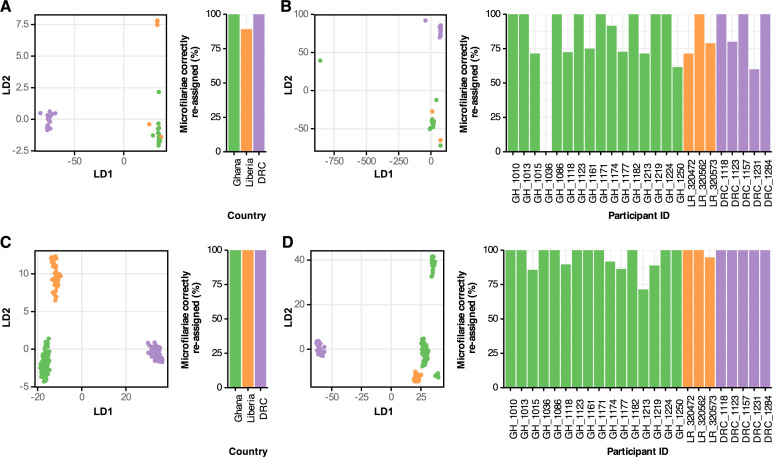
Genetic differentiation of *Onchocerca volvulus* microfilariae. (A) Discriminant Analysis of Principal Components (DAPC) based on the countries of origin and the proportions of successful reassignment using 206 mitochondrial genetic variants. (B) DAPC based on the hosts and the proportions of successful reassignment using 206 mitochondrial genetic variants. (C) DAPC based on the countries of origin and the proportions of successful reassignment using 9,227 nuclear genetic variants. (D) DAPC based on the hosts and the proportions of successful reassignment using 9,227 nuclear genetic variants.

We compared the results of Discriminant Analysis of Principal Components (DAPC) [40] using mitochondrial variants (n = 206) to those using nuclear variants (n = 9,227). For the mitochondrial data, the first 60 and 40 principal components were used for DAPC classification based on their country of origin and individual host, respectively (S3A and S3B Fig). For nuclear discriminant analyses, 4 and 60 principal components were used for genetic differentiation of mf based on their country of origin and individual host, respectively (S3C and S3D Fig). It was possible to genetically differentiate mf samples from West and Central Africa using both mitochondrial and nuclear variants, with a percentage of correct assignment to country of 96.4% and 100%, respectively (Fig 2A and 2C). However, the rate of correct reassignment of mf to their specific hosts based on their mitochondrial genotype was ≤ 80% for 11 out of 24 hosts (Ghana: 7 out of 16; Liberia: 2 out of 3 and DRC: 2 out of 5) and 83.6% overall (Fig 2B).

In contrast, nuclear data were more informative for the DAPC of mf by host. Five clusters can be observed in the plot: three associated with samples from Ghana, one with Liberia, and one with the DRC (Fig 2D). Most Ghana mf from the different hosts formed a single cluster, with the exception of mf from GH_1123 and GH_1224, which formed separate clusters, of which GH_1224 cluster was genetically the most distant. It was possible to differentiate mf populations from individual hosts with 100% accuracy for 17 of the 24 participants (Fig 2D). The mf populations from 23 out of 24 participants were successfully assigned to their respective host with more than 80% accuracy (Fig 2D). Overall, these analyses demonstrate that, using nuclear DNA variants, finer-scale differentiation of mf populations is possible between countries and between hosts within a country compared to more coarsely scaled differentiations based on mitochondrial DNA variants.

### Estimating the number of reproductively active adult parasites in participants based on the genetic diversity of offspring mf

To estimate reproductively active adult worm burden from mf data, we inferred parentage based on sibship reconstruction. We utilized nuclear genetic variants to compute genome-wide genetic relatedness in addition to mitochondrial, X-linked, and Y-linked haplotype sharing patterns between mf (S4–S6 Figs). Only hosts from which at least 9 mf had been sequenced were included in this analysis (Table 2). The number of reproductively active adult females estimated from the inferred maternal sibling groups was on average 4.0, 5.5, and 7.5 for Ghana (participants: n = 9), Liberia (n = 2), and DRC (n = 2), respectively (Table 2). The difference in estimates between the countries was not statistically significant (Kruskal–Wallis test, *P* = 0.22), although the small and unequal sample sizes limit the statistical power of this test. These estimates were lower than the number of unique mitochondrial haplotypes (Wilcoxon signed rank test, *P* = 0.048), which were 4.2, 6.0, and 10.5 for Ghana, Liberia, and the DRC, respectively (Table 2). In 6 out of the 62 identified maternal sibling families, mf with distinct mitochondrial haplotypes (differing by a single nucleotide substitution) were grouped into the same maternal family based on nuclear genetic relatedness, leading to many-to-one correspondences between mitochondrial haplotypes and inferred maternal sibling families, which contributed to the observed differences (S4 Fig and S3 Table). The rarefaction/extrapolation analysis of mf sampling depth showed estimated sample completeness ranging from 77% to 100%, with an average coverage of 92% (Table 2 and S7 Fig). This suggested that additional mf sampling would likely identify more maternal sibling families, particularly for hosts with lower coverage. To account for the potential under-detection of maternal sibling families due to limited sampling depth, we calculated asymptotic estimates along with their confidence intervals [41] (Table 2), yielding average adult female worm estimates of 6.6, 6.7, and 11.4 for Ghana, Liberia, and the DRC, respectively. In addition to the number of adult females, we estimated the number of reproductively active adult males by identifying distinct paternal lineages based on Y-linked variants (Table 2 and S6 Fig). The average number of paternal lineages per participant was 2.3, 4.5, and 5.0 for Ghana, Liberia, and the DRC, respectively (Table 2), which were lower than the inferred maternal lineages. Rarefaction/extrapolation analysis indicated a mean sample coverage of 95%, ranging from 76% to 100%, with asymptotic estimates of 2.4, 5.4, and 5.5 for Ghana, Liberia, and the DRC, respectively (Table 2 and S8 Fig).

**Table 2:** Estimates of *Onchocerca volvulus* adult worm counts based on the number of genetically inferred sibling families among microfilariae sampled from participants. Asymptotic estimates and sample coverage were calculated based on the Chao method [41].

Participant ID	Microfilariae sampled (female, male)	Number of unique mitochondrial haplotypes	Reproductively active adult worm estimation					
			Number of sibling families inferred from genomic data		Asymptotic estimate [95% confidence interval]		Estimated sample coverage	
			Adult female	Adult male	Adult female	Adult male	Adult female	Adult male
GH_1118	29 (17, 12)	10	9	5	23.5 [9.0, 40.9]	5.0 [5.0, 7.2]	80%	100%
GH_1123	12 (9, 3)	1	1	1	1.0 [1.0, 1.0]	1.0 [1.0, 1.0]	100%	100%
GH_1161	12 (5, 7)	6	5	2	7.8 [5.0, 12.2]	2.0 [2.0, 2.5]	77%	100%
GH_1171	10 (5, 5)	1	1	1	1.0 [1.0, 1.0]	1.0 [1.0, 1.0]	100%	100%
GH_1174	12 (6, 6)	4	4	2	5.8 [4.0, 10.4]	2.0 [2.0, 2.9]	85%	100%
GH_1177	22 (8, 14)	8	8	4	12.3 [8.0, 20.4]	4.0 [4.0, 5.6]	87%	100%
GH_1219	9 (4, 5)	3	3	3	3.0 [3.0, 3.9]	3.2 [3.0, 6.2]	100%	90%
GH_1224	19 (13, 6)	1	1	1	1.0 [1.0, 1.0]	1.0 [1.0, 1.0]	100%	100%
GH_1250	13 (6, 7)	4	4	2	4.0 [4.0, 5.3]	2.0 [2.0, 2.9]	100%	100%
LR_320472	14 (8, 6)	6	5	4	5.5 [5.0, 9.3]	4.8 [4.0, 9.9]	94%	76%
LR_320573	19 (8, 11)	6	6	5	7.9 [6.0, 11.9]	5.9 [5.0, 9.4]	90%	85%
DRC_1118	53 (26, 27)	10	5	4	5.0 [5.0, 5.2]	4.0 [4.0, 5.4]	100%	100%
DRC_1123	35 (20, 15)	11	10	6	17.8 [10.0, 36.9]	6.9 [6.0, 11.6]	89%	88%

### Comparison of adult worm estimates from mf genetic data with direct histological observations from nodules

**Fig 3:**
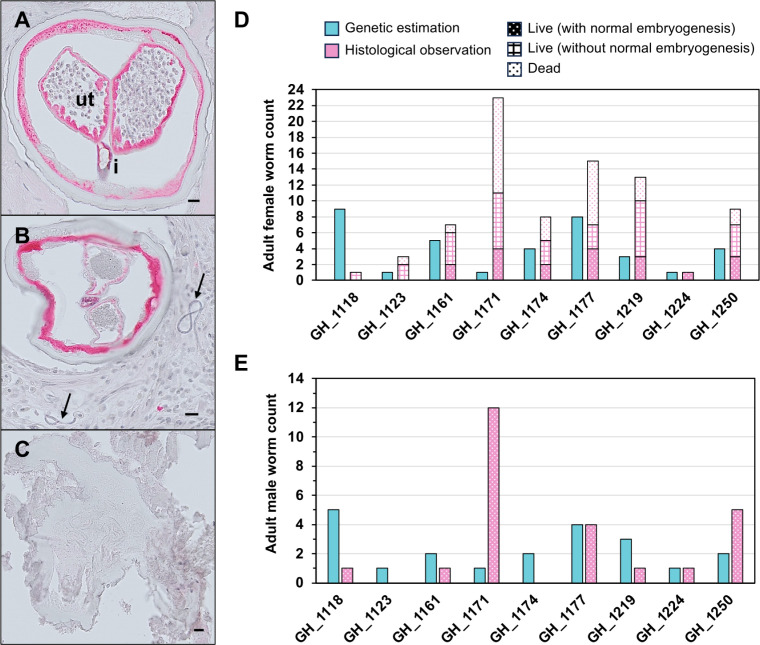
Comparison of *Onchocerca volvulus* adult worm estimates based on nodulectomy and genetic analysis of microfilariae (mf) for participants from Ghana. Histological evaluation of worm-sections stained with anti-aspartic protease sera (APR) for viability. (A) Adult female with normal embryogenesis. Stretched mf can be seen in uterus. Ut, uterus; i, intestine. (B) Adult female with degenerated embryos. Mf in nodule tissue (arrow). (C) Dead adult female worm. The number of adult worms estimated using mf genetic data and histological analysis of nodules: (D) Adult females, (E) Adult males.

The Ghana mf samples were collected from participants in a clinical study assessing the safety and efficacy of IDA treatment [42]. As part of the study, 18 month post-treatment nodules were surgically removed from study participants, and worm viability and fertility were assessed by histology (Fig 3A–3C). We compared our estimates of reproductive adult worm burden, based on the genetic analysis of skin mf, with those from the histological assessment of nodules from 9 participants in Ghana (Fig 3D, 3E and S4 Table). Both female and male adult worm counts were available for comparison. Female worms were further classified as live fertile worms with normal embryogenesis, live worms without normal embryogenesis, and dead worms (Fig 3A–3C). Genetics-based estimates were higher than or equal to live (fertile) worm counts from nodules in 8 of 9 individuals for female worms (Fig 3D) and in 7 of 9 for male worms (Fig 3E). These differences were statistically significant for adult females (Wilcoxon signed rank test, *P* = 0.036 for females and *P* = 0.057 for males). The correlation of counts was not significant in either females (R = 0.00, *P *= 1.00) or males (R = -0.21, *P* = 0.58) (S9 Fig). However, for the females, there were two substantial count outliers: GH_1118 and GH_1171. Without these two, the correlation between genetics-based estimates and viable fertile worm counts from nodules was higher (R = 0.83, *P* = 0.02). A similar pattern of correlation was observed when asymptotic estimates were used instead of the number of adults based on the number of inferred sibling families (Table 2 and S9 Fig).

### Comparing autosomal and X-linked genetic variation in *O. volvulus*

Using diploid genomes from individual mf samples, which are not affected by mixed haplotypes from embryonic material (as is the case with gravid adult female worms), we assessed genome-wide patterns of nucleotide diversity and linkage disequilibrium (LD) across the *O. volvulus* chromosomes. The Y chromosome was excluded from this analysis due to its incomplete assembly. To accurately estimate the level of genetic diversity and directly compare it between chromosomes, we performed the analysis using 28 unrelated female mf samples from 15 participants from a single country, Ghana. Male mf samples were excluded to prevent ploidy variations between autosomes and the X chromosome. Additionally, in male samples, short-read sequencing based variant calling cannot reliably differentiate between X-linked and Y-linked variants in reads aligned to the pseudoautosomal region (PAR) of the X chromosome.

**Fig 4:**
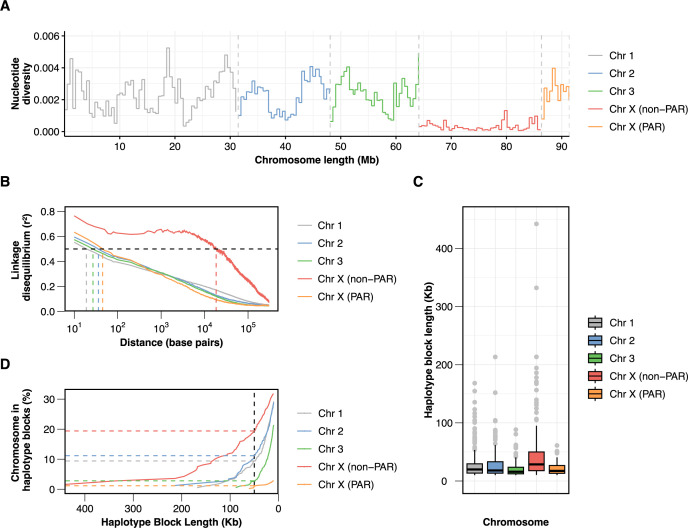
Genome-wide genetic diversity, linkage disequilibrium (LD), and haplotype blocks in *Onchocerca volvulus.* Chr 1 (OM1), 2 (OM3), 3 (OM4), and X (OM2) were analyzed using Ghanaian female microfilariae (n = 28). (A) Nucleotide diversity (π) in 500 kb sliding windows. (B) LD decay pattern. Vertical dotted lines indicate the distance at which r^2^ is 0.5 for each chromosome. (C) Box plot of haplotype block length distribution (>10 kb). (D) Proportion of chromosomes found within haplotype blocks. Horizontal dotted lines indicate the percentage of chromosomes found within ≥50 kb haplotype blocks.

A sliding window analysis of nucleotide diversity (π) revealed variation in diversity along the length of each chromosome, as well as between chromosomes (Fig 4A). The average nucleotide diversity was 0.0023, 0.0022, 0.0022, and 0.0007 for Chromosomes 1, 2, 3, and X, respectively. For the mitochondrial genome, the average nucleotide diversity among the analyzed samples was 0.028. Tajima’s D statistic was calculated to evaluate deviations from neutral evolution under equilibrium conditions [43]. The resulting values were -0.4, -0.5, -0.6, -0.9, and -2.3 for Chromosomes 1, 2, 3, X, and the mitochondrial genome, respectively. Within Chromosome X, nucleotide diversity was substantially lower in the non-PAR (22Mb, π = 0.0003) compared to the PAR (5.3Mb, π = 0.0025). The level of X-linked sequence diversity in the non-PAR relative to the autosomal genetic variation (π_X_/π_A_ = 0.14) was substantially lower than that expected for heteromorphic sex chromosomes (π_X_/π_A_ = 0.75 for a population with a 1:1 sex ratio, where the expected X chromosome effective population size (*N*_e_) is ¾ of the autosomal *N*_e_ due to the ploidy difference) (Wilcoxon rank sum test, *P* < 10^-10^). The level of X-linked sequence diversity in PAR was higher than that of the autosomes (π_X_/π_A_ = 1.14) (Wilcoxon rank sum test, *P* = 0.0017).

The average rate of LD decay was estimated using female mf from Ghana by the squared correlation coefficient (r^2^) for all pairs of SNPs within 300 kb in each chromosome (Fig 4B). On average, higher levels of LD were observed for SNPs in Chromosome X compared to those in the autosomes. Within Chromosome X, LD decayed at a slower rate in the non-PAR compared to the PAR. In the non-PAR, r^2^ decreased to 0.5 for SNPs that are 18.5 kb apart. In PAR, r^2^ decreased to 0.5 for SNPs that are 45 bp apart. We identified haplotype blocks, which are sizable regions with little evidence of historical recombination and within which only a few common haplotypes are observed [44]. The length distribution of haplotype blocks (>10 kb) for each chromosome displayed a pattern consistent with the variation in LD decay (Fig 4C). The median haplotype block lengths ranged between 15.9 kb and 19.9 kb for autosomes. For Chromosome X (non-PAR), the median haplotype block length was longer, approaching 28.6 kb. In addition, a larger proportion of the chromosome was found in longer haplotype blocks in Chromosome X (non-PAR) compared to the autosomes (Fig 4D).

## Discussion

In this study, we achieved high nuclear genome coverage through whole-genome amplification of *O. volvulus* mf, the larval stage routinely collected for diagnosis. Obtaining sufficient quantity and quality of DNA from single mf samples for comprehensive nuclear genome analysis has long been a challenge. Using our genome-wide nuclear genetic data from samples from Ghana, Liberia, and DRC, we have (i) examined the patterns of genetic structuring within and between hosts as well as between countries, (ii) estimated the number of reproductively active adult female and male worms in individual hosts, and (iii) assessed the genome-wide patterns of genetic variation across the different chromosomes in *O. volvulus*. Historically, the small physical size of mf (250–360 μm × 5–9 μm) [45], with an estimated <250 nuclei, and the variable (and often suboptimal) quality of sample preservation have limited the generation of high-quality sequencing data and full genomic analysis in *O. volvulus* mf. Using whole genome amplification with improved sample preservation, processing, and library construction for filarial nematodes that we have developed, validated and recently reported [35,46], we generated a WGS dataset (315 individual mf; 175, 37, and 103 samples from Ghana, Liberia, and DRC, respectively) with significantly improved nuclear genome coverage, achieving a median breadth of coverage of 94%. However, the quality of sequencing data varied between samples collected in different clinical trials conducted in different countries. Overall, nuclear genome coverage was higher for West African samples compared to DRC samples, likely due to variation in sample preservation. West African samples were stored in RNAlater at -20°C and shipped cold, while DRC samples were stored in ethanol and shipped at ambient temperature.

Analysis of genetic variation among mf samples can provide insights into population connectivity and historical gene flow useful for delineating transmission zones [34]. To date, mitochondrial genetic variation has been the primary genetic marker used in these population studies in *O. volvulus* [37–39]. Our dataset enabled, for the first time, a systematic comparison of patterns between nuclear and mitochondrial genetic variation in *O. volvulus* mf. Using autosomal SNPs that represent a large number of independent loci, it was possible to genetically differentiate parasite populations between hosts and between geographically close countries with higher resolution than mitochondrial haplotype-based analysis. Assigning individual worms to their source populations was more successful at both the individual host and country levels using allele-sharing patterns in the nuclear genome. These results indicate that, compared to mitochondrial data, nuclear genomic data (when combined with population sampling appropriate for the study aim) provide a higher resolution analysis that can capture more fine-grained genetic structure in the parasite population. Detecting such differences is essential for analyses aiming to detect shorter-distance parasite migration via host/vector movement. Compared to the mitochondrial genome, which is maternally inherited as a single locus (linkage group) without recombination, analysis of the sexually recombining nuclear genome can reveal admixture events and quantify the populations’ ancestry compositions. In addition, genome-wide analysis of nuclear genetic variation could provide deeper insight into the impact of MDA on parasite populations (e.g., selection of genotypes that provide survival/reproductive advantage in response to treatment), contributing to more effective and sustainable intervention strategies.

Genetic data from mf can be used to obtain information that is epidemiologically important but difficult to obtain through other methods, such as estimates of adult worm burden and fecundity in individual hosts [35,39]. The success of transmission interruption through MDA depends entirely on the cumulative decline in female fecundity over time due to repeated ivermectin treatments, but there is currently no direct method to measure this decline. While treatment does not reduce the number of palpable nodules, the presence of skin mf indicates that at least one fertile female remains, without providing a clear measure of progress toward sterility—information critical for assessing MDA progress and making decisions about when to stop treatment. Monitoring changes in reproductively active adult worm populations through longitudinal sampling of mf would help assess the effects of treatment, identify new infections, and detect treatment failures.

The first study using genetics to estimate the number of reproductive adults in *O. volvulus* by sequencing microfilariae was limited by using only mitochondrial data, which only represents reproductive females [39]. In this study, we estimated the number of both male and female reproductively active adults using nuclear genetic data and evaluated whether this approach improves estimates of worm burden. Our method for estimating adult worm counts from mf data was based on a sibship reconstruction approach previously used for *Wuchereria bancrofti* and *Brugia malayi*, the filarial species that cause lymphatic filariasis [35]. Sibling relationships were estimated using autosomal SNPs, and maternal or paternal sibling groups were assigned based on the segregation patterns of mitochondrial haplotypes, X-linked (excluding PAR), and Y-linked variants. We assessed whether microfilariae sampling was sufficient to detect the number of contributing parents using rarefaction and extrapolation analyses (S7 and S8 Figs) [35,39]. The estimated worm burden, and consequently the optimal number of microfilariae to sequence, varied between individuals and across countries (Figs 3 and S7), likely due to differences in age, exposure to infected blackflies based on time spent outdoors, and community-level transmission intensity. The number of maternal sibling families inferred from nuclear data, representing the number of reproductively active adult females, was lower than the number of unique mitochondrial haplotypes in 5 out of 13 participants. Given that mitochondrial DNA is maternally inherited in *O. volvulus*, we expected all maternal siblings within a host to share identical mitochondrial haplotypes. However, in 5 participants, mf with different mitochondrial haplotypes exhibited close autosomal relatedness, suggesting full or half-sibling relationships. Upon analyzing the genetic distance between these mitochondrial haplotypes, we found only a single nucleotide substitution, indicating possible genotyping errors. Consequently, these mitochondrial haplotypes were grouped into a single maternal family. To reduce the risk of erroneously merging distinct maternal sibling families (e.g., paternal half-siblings), we further examined the inheritance patterns of X-linked haplotypes among male mf to ensure consistency with the expectation that no more than two maternally inherited haplotypes segregate among maternal siblings. This cross-analysis of mitochondrial and X-linked haplotypes could also help identify cases where multiple adult females share the same mitochondrial haplotype, such as a high-frequency ancestral haplotype within the population.

Because whole-genome sequencing after DNA amplification using phi29 polymerase can increase genotyping errors [47], we applied stringent filtering to remove false-positive variants, including the exclusion of singleton SNPs prior to mitochondrial haplotype construction (see Methods for details). While determining the true genotyping error rate in amplified WGS data is challenging, these errors are likely to disproportionately affect nuclear and mitochondrial-based parentage inference. For sibship inference, a large number of independent SNPs were used to estimate genome-wide relatedness, making this analysis less vulnerable to genotyping errors introduced by genome amplification [35]. In contrast, the accuracy of mitochondrial haplotype determination is likely more sensitive to genotyping accuracy, as even a single substitution in the genome can result in distinct haplotypes. In addition to genotyping uncertainty, there are other possible explanations for the discordant patterns between mitochondrial and autosomal relatedness, such as inaccurate inference of sibling groups from autosomal data and unequal segregation of heteroplasmic mitochondrial genomes from an adult female to her progeny. Furthermore, identifying sibship groups through autosomal DNA comparison can be imprecise, as genetic relatedness is a continuous measure that may not perfectly align with theoretical expectations based on pedigree relationships. These variations depend on factors such as the number of chromosomes and their crossover rates [48].

An experimental approach to investigate the causes of these discrepancies would involve genotyping adult worms (somatic tissue) and *in utero* mf from nodules. Comparing the mitochondrial haplotypes of individual adult females to their intrauterine mf would be especially informative in determining whether multiple mitochondrial haplotypes can occur among the mf from a single female due to mitochondrial heteroplasmy. While we were unable to conduct this experiment, we compared genetic estimates of reproductively active adult worms with direct counts of adult worms from nodules extracted from 9 Ghanaian participants. We did not find a significant correlation between adult worm burden estimates based on mf genetic data and the fertile worm counts from nodules, although the genetic estimates were equal to or higher than the nodule worm counts in most cases. Accurately quantifying adult worm burden via nodulectomy can be difficult due to the presence of hidden nodules that cannot be palpated, as they may reside in deeper subcutaneous layers or within the body cavity [11–13]. Additionally, microfilariae can survive for approximately 18–24 months after being released by the female worm [11], making it possible that some of the genotyped mf in this study originated from infertile or deceased females that had stopped reproducing prior to nodulectomy. In 2 of the 9 participants, genetic estimates of adult males were possible despite no adult male worms being identified in the examined nodules. Since males migrate between nodules [14], they may not have been present in any nodules at the time the nodulectomies were performed. One limitation of our data is that we do not have pre- and post-treatment samples, and thus could not explicitly test some of the alternative hypotheses for ongoing transmission of *O. volvulus* in Ghana. However, we have recently shown that nuclear genome-based analysis of mf can effectively distinguish between recrudescence and reinfection in lymphatic filariasis [35], and we propose that similar approaches could be applied to *O. volvulus*.

Our study has significantly expanded the number of nuclear genomes available for population genomic analysis, increasing the dataset by more than 300 genomes, compared to the ~ 30 previously sequenced at comparable depth and coverage [36,37,39]. This new nuclear genome data, derived from individual single mf, enabled the first accurate comparison of genetic variation between sex chromosomes and autosomes in *O. volvulus*, without the confounding effects of embryonic DNA that can compromise the genotyping accuracy of maternal DNA in adult worm sequencing. Using 28 unrelated female mf samples (≥ third-degree kinship) from Ghana, we analyzed patterns of nucleotide diversity (π) and LD across the genome. Our genome-wide estimate of nucleotide diversity in the nuclear genome (π = 2 × 10^-3^) was an order of magnitude lower than that of the mitochondrial genome (π = 3 × 10^-2^) and lower than our previous global estimate (π = 4 × 10^-3^) [36], yet notably higher than values reported for other vector-borne filarial parasites, *Wuchereria bancrofti* (π = 2 × 10^-4^) [49] and *Dirofilaria immitis* (π = 4 × 10^-5^ to 7 × 10^-4^) [50]. Negative Tajima’s D values were observed across both the nuclear and mitochondrial genomes, indicating an excess of low-frequency polymorphisms relative to expectations under neutrality. This pattern may reflect recent population expansion following a bottleneck. Our findings revealed lower than expected sequence diversity and extended LD in the non-PAR region of the X chromosome, compared to the PAR and autosomes. Comparative genomic analyses of filarial nematode species indicate that a recent X-autosome fusion event led to neo-X/Y formation in the last common ancestor of *Dirofilaria* and *Onchocerca* [51,52]. This fusion likely altered recombination patterns, suppressing it near the fusion point and resulting in a loss of genetic diversity [53,54]. Low X-linked genetic diversity appears to be a hallmark of filarial species that have undergone neo-X/Y formation [51].

Patterns of LD decay provide valuable insights into historical recombination events. LD is influenced by both the recombination rate and the number of generations that have undergone recombination [55]. Our evidence suggests that the X chromosome in *O. volvulus* likely experiences a lower recombination rate than the rest of the genome, making it more vulnerable to Muller’s Ratchet [56,57], a process that leads to the stochastic accumulation of mildly deleterious mutations over time. Additionally, recessive variants under positive or purifying selection are expected to go to fixation more rapidly on the X chromosome due to hemizygosity in males, who carry only one copy of the X chromosome. This makes genes in the non-PAR more “exposed” to selection [58], potentially reducing genetic variation across large portions of the X chromosome. Given that genetic diversity is a key determinant of a population’s ability to adapt to changing environmental conditions, including exposure to anthelmintics or different vector species, the reduced genetic diversity on the X chromosome, which accounts for ~28% of the *O. volvulus* genome, may have significant evolutionary consequences.

In conclusion, our findings suggest that fine-scale genetic structure in *O. volvulus* populations can be detected using mf whole-genome nuclear data, offering greater resolution than previous studies that used mitochondrial data alone. Additionally, we applied sibship reconstruction with autosomal and sex chromosomal SNPs to estimate the minimum number of reproductively active adult females and males in individual hosts. This work lays the groundwork for developing a genetic approach that could provide invaluable information for the MDA program. Furthermore, our whole-genome data enabled a detailed analysis of genome-wide genetic variation, confirming lower-than-expected genetic diversity on the X chromosome. This study contributes to the application of modern population genomics tools in support of onchocerciasis elimination efforts.

## Methods

### Ethics

*Onchocerca volvulus* mf were obtained from infected participants in Liberia as part of a clinical trial (ClinicalTrial.gov Identifier: NCT01905436) [59] and in Ghana during a trial evaluating the efficacy of a triple-drug regimen (ClinicalTrials.gov Identifier: NCT04188301) [42]. Mf from the Democratic Republic of the Congo (DRC) were obtained from a clinical trial run by the WHO where the use of ivermectin and moxidectin for MDA are being compared (MDGH-MOX-3001 and MDGH-MOX-3002). Genome sequencing of mf from de-identified human participants received a full waiver of HIPAA Authorization (IRB ID #:201910085), and non-human subjects’ determination by the Washington University in St. Louis Institutional Review Board (DHHS Federalwide Assurance #FWA00002284). Committee approval for both clinical studies in DRC were obtained from the Comité National d’Ethique de la Santé (CNES) of DRC (N 204/CNES/BN/PMMF/2020, 26 August 2020) and the WHO Ethics Review Committee (MDGH-MOX-3001 28 September 2020, MDGH-MOX-3002 29 September 2020).

### Parasite material

Mf were collected from 24 participants from Ghana (n = 16), Liberia (n = 3) and the DRC (n = 5) (S1 Table). Participants from Liberia were drug-naïve, and those from the DRC had received no or very few prior ivermectin treatments. The mf samples from Ghana were collected from treated participants 18 months post-treatment (S1 Table). The skin snips collected from Ghana and Liberia were incubated overnight in physiological saline solution at ambient temperature. Following incubation, RNAlater was added, and the entire contents were stored at -20°C for shipment to Washington University in Saint Louis (Missouri, USA) for downstream processing. The mf were picked and placed into a 1.5 ml tube containing 1x PBS, followed by 3–4 washing steps. Finally, a volume of 200 µl 1x PBS was screened for single mf under a microscope. Individual mf were picked at 4-fold magnification using a 10 µl pipettor, and mf in 2μl of 1x PBS was transferred into a 0.2 ml microfuge tube. This step was followed by either the isolation of genomic DNA or freezing at -20°C for further use. The collection and storage of skin snips from the DRC were performed as previously described [39,46]. In brief, four skin snips were obtained from each participant in two trials (MDGH-MOX-3001 and MDGH-MOX-3002). These snips were incubated in physiological saline to allow mf to emerge for counting. The contents of the wells (mf + saline) were then added to ethanol to achieve a final concentration of ≥74%, and tubes were shipped to La Trobe University (Victoria, Australia). For DNA lysates prepared at La Trobe University, single mf were picked from the ~ 70% v/v ethanol solution into MilliQ water and then into 10μl of DNA lysis buffer using an eyelash or fine platinum-iridium wire.

### DNA isolation and qPCR-based detection of *O. volvulus* DNA

For the isolation of genomic DNA from single mf, 25 µl of lysis buffer (950 µl nuclease-free water, 30 µl 3 M Tris-HCL, 5 µl NP-40, 5 µl Tween-20, and 10 µl Proteinase K) was added to each tube containing single mf. The samples were then incubated at 55°C for 2 hours, followed by a 20 min incubation at 85°C for proteinase K inactivation and then stored at 4°C till further processing. Isolation of genomic DNA from the ethanol preserved mf from DRC were carried out using the same conditions but in a volume of 10 μl of DNA lysis buffer. Presence of *O. volvulus* DNA in samples was confirmed by qPCR targeting 128 bp portion of ND5 gene of *O. volvulus* (Forward primer: 5′-GCTATTGGTAGGGGTTTGCAT-3′, Reverse primer: 5’-CCACGATAATCCTGTTGACCA-3’ and Probe: 5′-FAM TAAGAGGTTAAGATGG NFQ-3′) [60].

### Whole genome amplification and sequencing of microfilariae

Amplification of isolated genomic DNA from individual mf was carried out using the Ready-To-Go GenomiPhi V3 DNA Amplification kit (Cytiva, Marlborough, MA). Human DNA contamination was quantified using qPCR targeting a section of Chromosome I, as described previously (forward primer: 5’-ACTTTGGGTCATTCCCACTG-3’, and reverse primer: 5’-GCTCAGCTCCTTGCTGGATA-3’) [49]. For each sample with a yield greater than 1 µg, the total yield generated after whole genome amplification was used for library construction and whole genome sequencing (WGS). Library construction and indexing of samples were carried out using the KAPA hyper PCR-free kit and the KAPA unique dual-indexed adapter kit, respectively. Libraries were validated with qPCR using the KAPA library quantification kit and sequenced on Illumina’s NovaSeq platform (2 × 150 bp paired end reads), targeting 10Gb per sample, by the McDonnell Genome Institute at Washington University in Saint Louis (Missouri, USA).

### Processing of WGS data and variant calling

The sequencing data were assessed using FastQC v0.11.8 [61], followed by removal of adaptors and base sequences with low quality scores using Trimmomatic v0.39 (ILLUMINACLIP:TruSeq3-PE.fa:2:30:10:2 SLIDINGWINDOW:4:15 MINLEN:36) [62]. Trimmed reads were mapped to a combined database containing reference genome sequences of the nuclear and mitochondrial genomes of *O. volvulus* (V4; James Cotton, personal communication, Feb 4, 2020) [63], the *Wolbachia* endosymbiont (GenBank: NZ_HG810405.1) and *Homo sapiens* (GenBank: GCA_000001405.28) using BWA v0.7.17 [63,64]. Picard v2.27.5 (http://broadinstitute.github.io/picard/) was used to sort, add read group information, and remove PCR/optical duplicates. The sex of each individual mf sample was determined based on the ratio of total read counts mapping to the X-chromosome and autosomes, and the breadth of Y-chromosome coverage (percentage covered by at least 10X). Individual mf were identified as male if the normalized read count ratio was ~ 0.5 and Y-chromosome coverage was more than 10%. Variant calling on the nuclear genome of *O. volvulus* was performed for each sample using GATK v4.3.0.0 HaplotypeCaller [65] in Genomic Variant Call Format (GVCF) mode with the minimum mapping parameter set at 30. Multisample genotyping was performed using GATK CombineGVCFs and GenotypeGVCFs. SNPs were extracted using GATK SelectVariants and filtered using GATK VariantFiltration with the following settings: QD (variant confidence normalized by unfiltered depth of variant samples) < 2.0; QUAL (read quality) < 30.0; FS (strand bias estimated using Fisher’s exact test) > 60.0; MQ (Root mean square of the mapping quality of reads across all samples) < 40.0; SOR (Strand bias estimated by the symmetric odds ratio test) > 3.0; MQRankSum (Rank sum test for mapping qualities of REF versus ALT reads) <-10.0; ReadPosRankSum (Rank sum test for relative positioning of REF versus ALT alleles within reads) <-10.0; ReadPosRankSum > 10.0 and DP (maximum depth)> (median depth x 2). VCFtools v0.1.16 [66] was used to calculate missing data proportion, heterozygosity, and inbreeding coefficients for each sample. Samples with genotype missingness greater than or equal to 11% or negative inbreeding coefficient values less than -0.05 (indicative of possible sample contamination) were excluded from downstream analysis. The command-line arguments used in the analysis are provided in S1 Text.

### Variant calling on mitochondrial DNA and construction of haplotype network

Variant calling on mitochondrial DNA (mtDNA) of *O. volvulus* was conducted using GATK v4.3.0.0 HaplotypeCaller with the following parameters: --linked-de-bruijn-graph, --sample-ploidy 1, --minimum-mapping-quality 30, and -ERC GVCF. Final variants were called on consolidated GVCF using GATK GenotypeGVCFs. GATK SelectVariants was used to extract SNPs and further sites filtering was performed using GATK VariantFiltration with following settings: QD < 2.0; QUAL < 30.0; FS > 60.0; MQ < 40.0; SOR > 3.0; MQRankSum < -10.0; ReadPosRankSum < -10.0; ReadPosRankSum > 10.0; DP < 20.0. To further remove false-positive variant loci, SNP sites with heterozygous genotype calls were identified by running GATK with --sample-ploidy 2. These problematic sites were then removed from subsequent analysis. After removing singleton SNPs, the VCF file was converted to tabular format using vcftool v0.1.16, followed by conversion to multi-fasta format using trimal v1.4.1 [67]. Subsequently, a mitochondrial haplotype network was constructed using the TCS algorithm with PopArt v1.7 [68]. At this step, samples with more than 5% missing variant sites were excluded.

### Discriminant analysis of principal component (DAPC) analysis

DAPC [40] was carried out using *adegenet* v2.1.10 [69] to construct discriminant functions that maximize the variance components between predefined groups, such as the countries of origin and the hosts (participants). Using cross-validation, the number of principal components to be used in DAPC was determined to avoid under/overfitting of the model. Prior to the analysis, autosomal SNPs were pruned to remove variants that are in strong LD using PLINK v1.90 [70] (--maf 0.05 --indep-pairwise 200 5 0.2 --geno 0.01). DAPC was performed using either autosomal and mitochondrial variants to differentiate mf populations from different countries and hosts, and the accuracy of the group assignment was compared between the two marker types.

### Sibship reconstruction and parentage inference

Genetic sibship reconstruction provides a method for estimating adult worm burden and identifying surviving worm families post-treatment. This study utilized two types of genetic data, nuclear and mitochondrial, to assess relatedness and reconstruct sibships from mf isolated from infected individuals. Because mitochondrial DNA is maternally inherited in *O. volvulus*, the number of unique mitochondrial haplotypes detected within infected participants serves as a minimum estimate of reproductively active adult females. However, the sensitivity of this method may be limited by the genetic diversity of mitochondrial haplotypes in the population. If common haplotypes are shared among adult females within a host, the number of unique haplotypes identified among mf will underestimate the true number of reproductively active adult females. Additionally, genotyping errors associated with DNA amplification and the resulting uncertainty in haplotype determination can complicate efforts to identify the exact number of unique haplotypes, particularly when haplotypes differ by only a single nucleotide substitution.

After variant filtering in PLINK v1.90 (--mac 4 --geno 0.05), we used genome-wide autosomal SNPs to estimate kinship coefficients and infer familial relatedness among mf within each host using PC-Relate, implemented in GENESIS v2.34 [71]. The resulting genetic relatedness matrix was reordered using the average linkage method in ggcorrplot v0.1.4.1 [72] to cluster mf samples into groups of closely related individuals and to evaluate the distribution of mitochondrial haplotypes across these groups. After confirming the expected correspondence between autosomal relatedness and the grouping of mitochondrial haplotypes, we assigned mf into maternal sibling families based on mitochondrial haplotypes, with one exception. When mf with different mitochondrial haplotypes were grouped together based on their autosomal relatedness, we examined the genetic distance between the haplotypes. If the distance was only a single nucleotide substitution, we grouped the mitochondrial haplotypes into a single maternal family. To minimize erroneous grouping of maternal sibling families, we also evaluated the inheritance pattern of X-linked haplotypes among the male mf to ensure consistency with the expectation that at most two maternally inherited haplotypes (along with their recombinants) segregate among maternal siblings. To determine X-linked haplotypes in male mf, we constructed maximum-likelihood (ML) phylogenetic trees using IQ-TREE v1.6.12 [73], with ModelFinder [73] automatically selecting the best-fit model. This analysis used X-linked SNPs after removing loci with heterozygous genotype calls in bcftools v1.9 (-g ^het --min-ac 4:minor) [74] and loci with missing genotype calls in PLINK v1.9 (--geno 0). TreeCluster v1.0.4 [75] (--method max) was subsequently used to identify clusters of repeatedly observed sequences with significant similarity (--threshold 0.015, 0.03, and 0.02 for Ghana, Liberia, and DRC, respectively) and classify them into haplotypes. Following this clustering procedure, singleton sequences that remained indicated either (i) a low-frequency haplotype sampled only once or (ii) a recombinant haplotype, which could be unique due to variations in meiotic recombination breakpoints within gametes. To differentiate the former from the latter, only sequences that were substantially different from all other haplotypes (--threshold 0.035, 0.09, and 0.06 for Ghana, Liberia, and DRC, respectively) were classified as singleton haplotypes. Paternal sibling groups were identified based on the inheritance pattern of Y-linked haplotypes in male mf. To identify Y-linked haplotypes, ML phylogenetic trees were constructed using Y-linked SNPs, and sequences with significant similarity were identified by TreeCluster (--method max; --threshold 0.0005, 0.015 and 0.001 for Ghana, Liberia, and DRC, respectively). Rarefaction analysis was performed using iNEXT v3.0.0 [76] to assess the effect of mf sample size on the estimated number of adult male and female worms and to derive asymptotic estimates with their associated standard errors [77,78].

### Analysis of nucleotide diversity and patterns of linkage disequilibrium in *O. volvulus* genome

Only mf sequences from Ghana were used for these analyses, as the sample sizes for Liberia and DRC were too small. To compare patterns of genetic diversity across both the autosomes and the X chromosome, only female samples were used to avoid ploidy differences between the autosomes and the X chromosome, and to prevent Y-linked sequences that map to the pseudo-autosomal region (PAR) of the X chromosome from confounding genotyping in the region. After removing closely related samples (in second degree or closer kinship) using PLINK v2.00 (--king-cutoff 0.0884) [70], nucleotide diversity (π) was calculated in 10 kb sliding windows with pixy v1.2.7 [79] with its default settings. For visualization, sliding windows of 500 kb were used. The decay pattern of linkage disequilibrium (LD) was assessed and visualized using PopLDdecay v3.42 (-MAF 0.05) [55]. In chromosome X, LD decay was calculated for the PAR and non-PAR separately. Haplotype blocks were inferred from the same set of samples using PLINK v1.90 (--blocks no-pheno-req --blocks-max-kb 1000) following the block definition used in Haploview [44].

## Supporting information

S1 Fig Distribution of male and female microfilariae among 315 sequenced samples across all 24 participants. (PDF)

S2 Fig *Onchocerca volvulus* mitochondrial haplotype networks based on 206 SNP variants. (PDF)

S3 Fig Cross-validation for determining the optimal number of principal components to be retained for DAPC. (PDF)

S4 Fig Average linkage clustering of microfilariae based on kinship coefficients estimated using autosomal SNPs. (PDF)

S5 Fig Maximum likelihood phylogenetic tree of male microfilariae based on X-linked SNPs. (PDF)

S6 Fig Maximum likelihood phylogenetic tree of male microfilariae based on Y-linked SNPs. (PDF)

S7 Fig Rarefaction and extrapolation curves for *Onchocerca volvulus* maternal sibling families identified from hosts with microfilariae count of 9 or more. (PDF)

S8 Fig Rarefaction and extrapolation curves for *Onchocerca volvulus* paternal sibling families identified from hosts using male microfilariae. (PDF)

S9 Fig Correlation between genetically estimated and histologically observed *Onchocerca volvulus* adult worm counts for participants from Ghana. (PDF)

S1 Table Anthelmintic treatment history for each participant and microfilariae density pre- and post- treatment. (PDF)

S2 Table Single microfilaria whole genome sequencing data, including read alignment and genome coverage statistics, sample sex determination, genotype missingness, and inbreeding coefficients. (XLSX)

S3 Table Maternal and paternal sibling family inference based on autosomal relatedness, mitochondrial, and sex-linked haplotypes. (XLSX)

S4 Table Comparison of the number of adult worms estimated from microfilariae genetic data and those identified through histological analysis of nodules. (PDF)

S1 Text Bioinformatics pipeline and command-line arguments used in the analysis. (PDF)
