# Supplementary material for: Genetic structuring and estimation of reproductive adults in Onchocerca volvulus: A genome-wide analysis across hosts and regions
Source: PLoS Negl Trop Dis. 2025 Jul 1;19(7):e0013221. doi: 10.1371/journal.pntd.0013221 (PMC12212510; doi:10.1371/journal.pntd.0013221)
Supplement: S1 Fig — (PDF) [file pntd.0013221.s001.pdf]

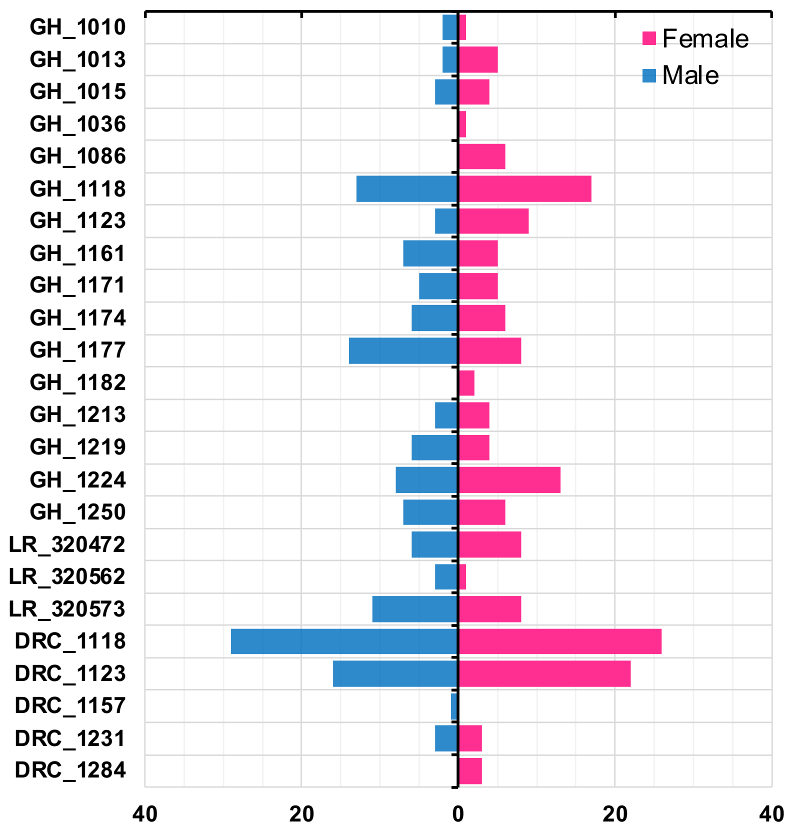

S1 Fig. Distribution of male and female microfilariae among 315 sequenced samples across all 24 participants.
