# Supplementary material for: Genetic structuring and estimation of reproductive adults in Onchocerca volvulus: A genome-wide analysis across hosts and regions
Source: PLoS Negl Trop Dis. 2025 Jul 1;19(7):e0013221. doi: 10.1371/journal.pntd.0013221 (PMC12212510; doi:10.1371/journal.pntd.0013221)
Supplement: S2 Fig — (PDF) [file pntd.0013221.s002.pdf]

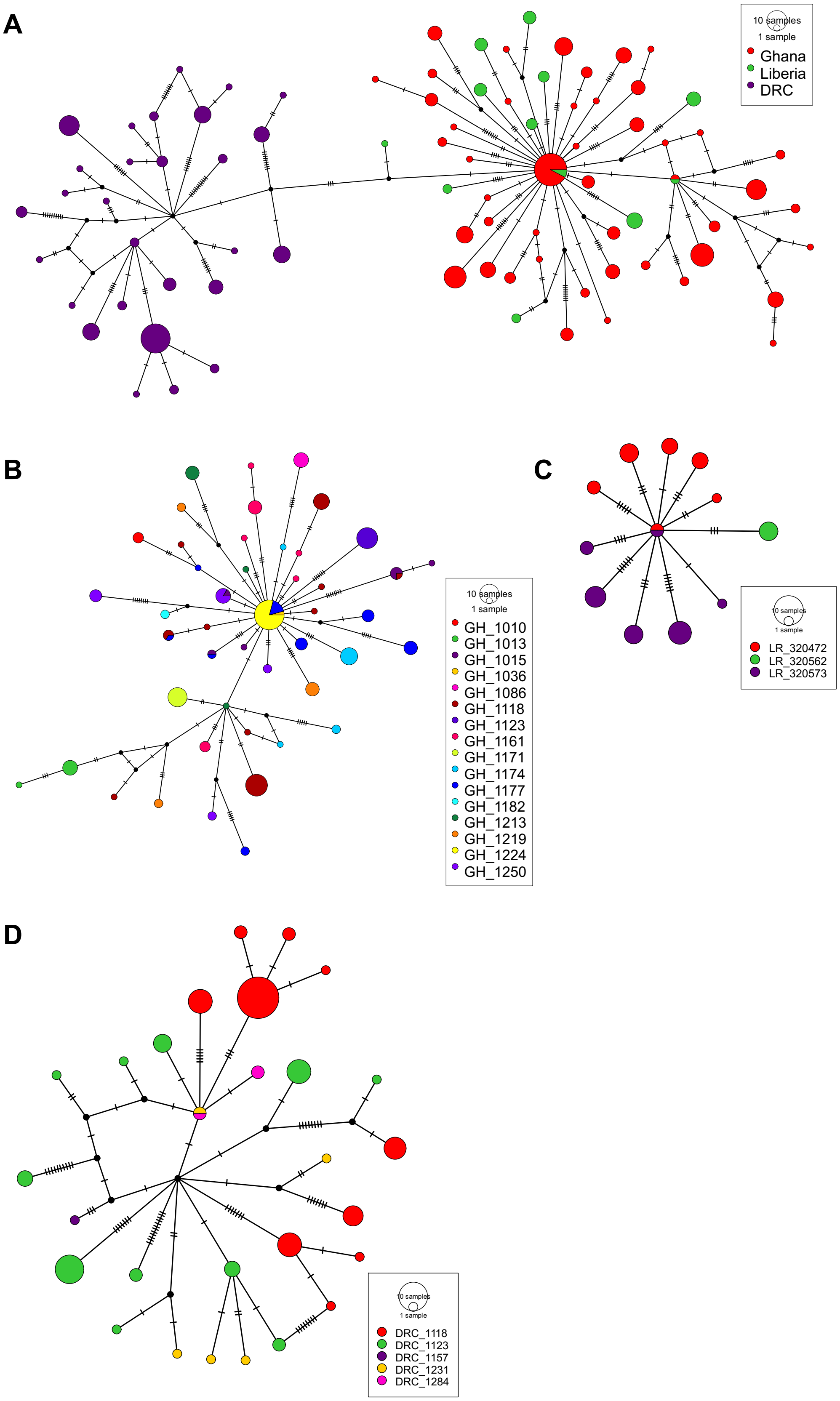

S2 Fig. *Onchocerca volvulus* mitochondrial haplotype networks based on 206 SNP variants. (A) Haplotype network of 305 microfilariae from 24 participants in Ghana, Liberia and the DRC. (B) Haplotype network of 171 mf from 16 participants in Ghana. (C) Haplotype network of 37 mf from 3 participants in Liberia. (D) Haplotype network of 97 microfilariae from 5 participants in the DRC. Each circle represents a unique haplotype, colored based on the country or individual participant; the size of the circle reflects the number of sequences. Hatch marks on the connecting lines indicate the number of nucleotide differences between haplotypes.
