# Supplementary material for: Genetic structuring and estimation of reproductive adults in Onchocerca volvulus: A genome-wide analysis across hosts and regions
Source: PLoS Negl Trop Dis. 2025 Jul 1;19(7):e0013221. doi: 10.1371/journal.pntd.0013221 (PMC12212510; doi:10.1371/journal.pntd.0013221)
Supplement: S3 Fig — (PDF) [file pntd.0013221.s003.pdf]

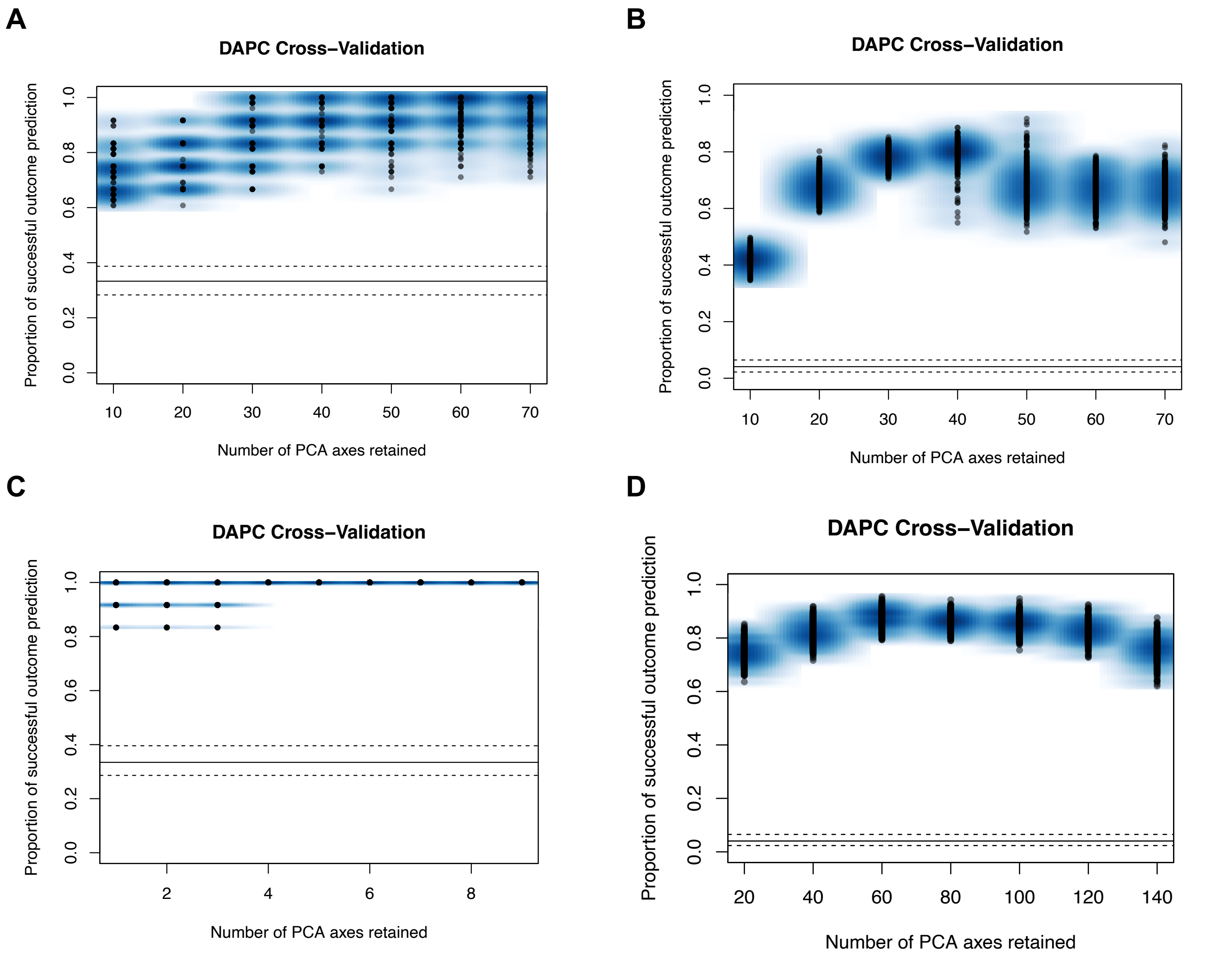

S3 Fig. Cross-validation for determining the optimal number of principal components to be retained for DAPC. (A) Mitochondrial variants-based analysis of 3 predefined groups by country. (B) Mitochondrial variants-based analysis of 24 predefined groups by host. (C) Nuclear variants-based analysis of 3 predefined groups by country. (D) Nuclear variants-based analysis of 24 predefined groups by host.
