# Supplementary material for: Genetic structuring and estimation of reproductive adults in Onchocerca volvulus: A genome-wide analysis across hosts and regions
Source: PLoS Negl Trop Dis. 2025 Jul 1;19(7):e0013221. doi: 10.1371/journal.pntd.0013221 (PMC12212510; doi:10.1371/journal.pntd.0013221)
Supplement: S4 Fig — (PDF) [file pntd.0013221.s004.pdf]

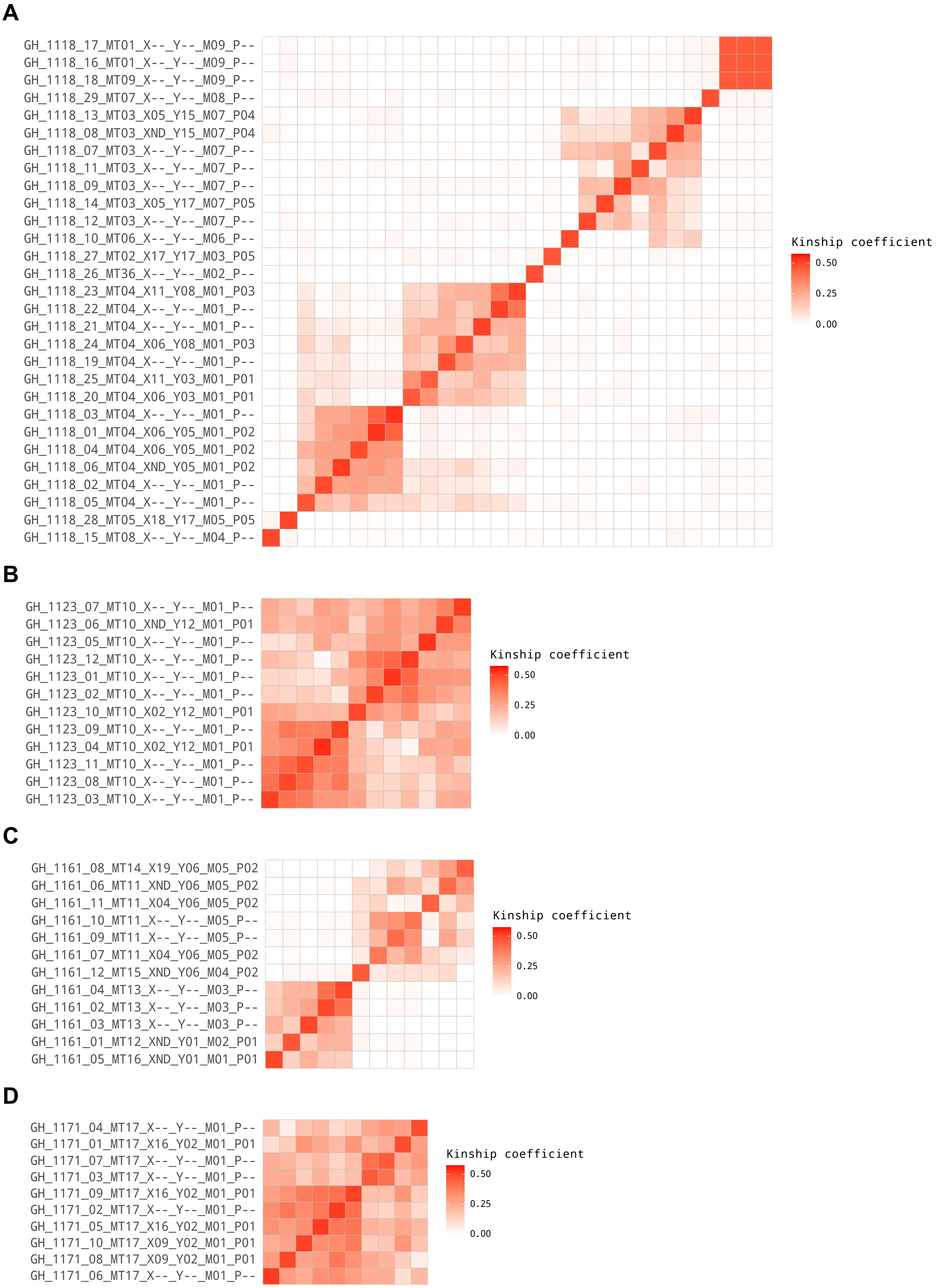
A
GH_1118_17_MT01_X--_Y--_M09_P--
GH_1118_16_MT01_X--_Y--_M09_P--
GH_1118_18_MT09_X--_Y--_M09_P--
GH_1118_29_MT07_X--_Y--_M08_P--
GH_1118_13_MT03_X05_Y15_M07_P04
GH_1118_08_MT03_XND_Y15_M07_P04
GH_1118_07_MT03_X--_Y--_M07_P--
GH_1118_11_MT03_X--_Y--_M07_P--
GH_1118_09_MT03_X--_Y--_M07_P--
GH_1118_14_MT03_X05_Y17_M07_P05
GH_1118_12_MT03_X--_Y--_M07_P--
GH_1118_10_MT06_X--_Y--_M06_P--
GH_1118_27_MT02_X17_Y17_M03_P05
GH_1118_26_MT36_X--_Y--_M02_P--
GH_1118_23_MT04_X11_Y08_M01_P03
GH_1118_22_MT04_X--_Y--_M01_P--
GH_1118_21_MT04_X--_Y--_M01_P--
GH_1118_24_MT04_X06_Y08_M01_P03
GH_1118_19_MT04_X--_Y--_M01_P--
GH_1118_25_MT04_X11_Y03_M01_P01
GH_1118_20_MT04_X06_Y03_M01_P01
GH_1118_03_MT04_X--_Y--_M01_P--
GH_1118_01_MT04_X06_Y05_M01_P02
GH_1118_04_MT04_X06_Y05_M01_P02
GH_1118_06_MT04_XND_Y05_M01_P02
GH_1118_02_MT04_X--_Y--_M01_P--
GH_1118_05_MT04_X--_Y--_M01_P--
GH_1118_28_MT05_X18_Y17_M05_P05
GH_1118_15_MT08_X--_Y--_M04_P--
Kinship coefficient
0.50
0.25
0.00
B
GH_1123_07_MT10_X--_Y--_M01_P--
GH_1123_06_MT10_XND_Y12_M01_P01
GH_1123_05_MT10_X--_Y--_M01_P--
GH_1123_12_MT10_X--_Y--_M01_P--
GH_1123_01_MT10_X--_Y--_M01_P--
GH_1123_02_MT10_X--_Y--_M01_P--
GH_1123_10_MT10_X02_Y12_M01_P01
GH_1123_09_MT10_X--_Y--_M01_P--
GH_1123_04_MT10_X02_Y12_M01_P01
GH_1123_11_MT10_X--_Y--_M01_P--
GH_1123_08_MT10_X--_Y--_M01_P--
GH_1123_03_MT10_X--_Y--_M01_P--
Kinship coefficient
0.50
0.25
0.00
C
GH_1161_08_MT14_X19_Y06_M05_P02
GH_1161_06_MT11_XND_Y06_M05_P02
GH_1161_11_MT11_X04_Y06_M05_P02
GH_1161_10_MT11_X--_Y--_M05_P--
GH_1161_09_MT11_X--_Y--_M05_P--
GH_1161_07_MT11_X04_Y06_M05_P02
GH_1161_12_MT15_XND_Y06_M04_P02
GH_1161_04_MT13_X--_Y--_M03_P--
GH_1161_02_MT13_X--_Y--_M03_P--
GH_1161_03_MT13_X--_Y--_M03_P--
GH_1161_01_MT12_XND_Y01_M02_P01
GH_1161_05_MT16_XND_Y01_M01_P01
Kinship coefficient
0.50
0.25
0.00
D
GH_1171_04_MT17_X--_Y--_M01_P--
GH_1171_01_MT17_X16_Y02_M01_P01
GH_1171_07_MT17_X--_Y--_M01_P--
GH_1171_03_MT17_X--_Y--_M01_P--
GH_1171_09_MT17_X16_Y02_M01_P01
GH_1171_02_MT17_X--_Y--_M01_P--
GH_1171_05_MT17_X16_Y02_M01_P01
GH_1171_10_MT17_X09_Y02_M01_P01
GH_1171_08_MT17_X09_Y02_M01_P01
GH_1171_06_MT17_X--_Y--_M01_P--
Kinship coefficient
0.50
0.25
0.00

E

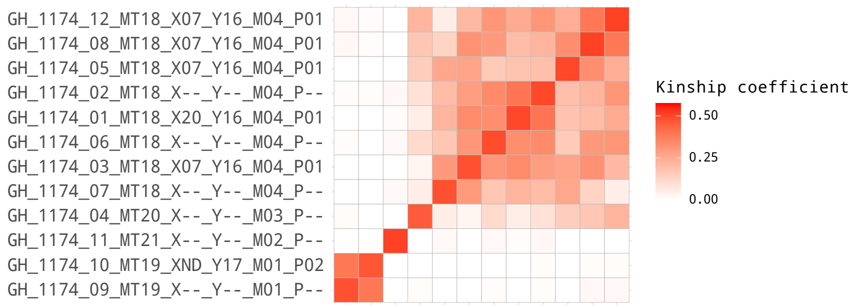
GH_1174_12_MT18_X07_Y16_M04_P01
GH_1174_08_MT18_X07_Y16_M04_P01
GH_1174_05_MT18_X07_Y16_M04_P01
GH_1174_02_MT18_X--_Y--_M04_P--
GH_1174_01_MT18_X20_Y16_M04_P01
GH_1174_06_MT18_X--_Y--_M04_P--
GH_1174_03_MT18_X07_Y16_M04_P01
GH_1174_07_MT18_X--_Y--_M04_P--
GH_1174_04_MT20_X--_Y--_M03_P--
GH_1174_11_MT21_X--_Y--_M02_P--
GH_1174_10_MT19_XND_Y17_M01_P02
GH_1174_09_MT19_X--_Y--_M01_P--
Kinship coefficient
0.50
0.25
0.00

F

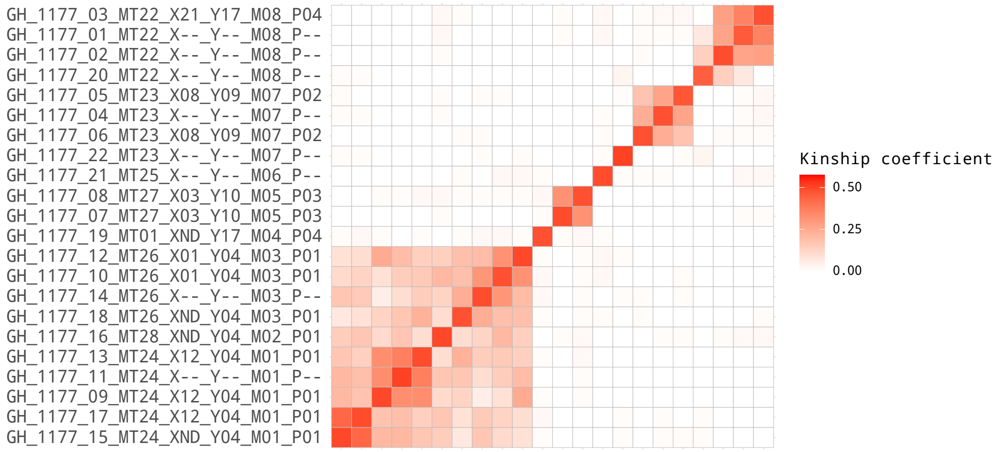
GH_1177_03_MT22_X21_Y17_M08_P04
GH_1177_01_MT22_X--_Y--_M08_P--
GH_1177_02_MT22_X--_Y--_M08_P--
GH_1177_20_MT22_X--_Y--_M08_P--
GH_1177_05_MT23_X08_Y09_M07_P02
GH_1177_04_MT23_X--_Y--_M07_P--
GH_1177_06_MT23_X08_Y09_M07_P02
GH_1177_22_MT23_X--_Y--_M07_P--
GH_1177_21_MT25_X--_Y--_M06_P--
GH_1177_08_MT27_X03_Y10_M05_P03
GH_1177_07_MT27_X03_Y10_M05_P03
GH_1177_19_MT01_XND_Y17_M04_P04
GH_1177_12_MT26_X01_Y04_M03_P01
GH_1177_10_MT26_X01_Y04_M03_P01
GH_1177_14_MT26_X--_Y--_M03_P--
GH_1177_18_MT26_XND_Y04_M03_P01
GH_1177_16_MT28_XND_Y04_M02_P01
GH_1177_13_MT24_X12_Y04_M01_P01
GH_1177_11_MT24_X--_Y--_M01_P--
GH_1177_09_MT24_X12_Y04_M01_P01
GH_1177_17_MT24_X12_Y04_M01_P01
GH_1177_15_MT24_XND_Y04_M01_P01
Kinship coefficient
0.50
0.25
0.00

G

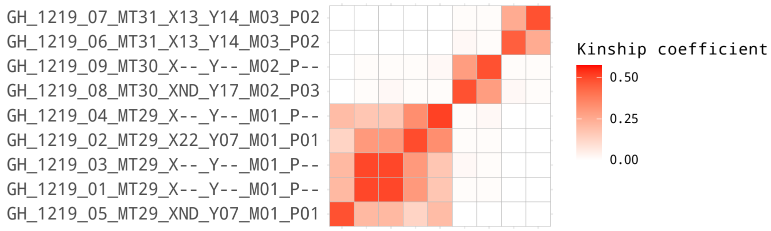
GH_1219_07_MT31_X13_Y14_M03_P02
GH_1219_06_MT31_X13_Y14_M03_P02
GH_1219_09_MT30_X--_Y--_M02_P--
GH_1219_08_MT30_XND_Y17_M02_P03
GH_1219_04_MT29_X--_Y--_M01_P--
GH_1219_02_MT29_X22_Y07_M01_P01
GH_1219_03_MT29_X--_Y--_M01_P--
GH_1219_01_MT29_X--_Y--_M01_P--
GH_1219_05_MT29_XND_Y07_M01_P01
Kinship coefficient
0.50
0.25
0.00

H

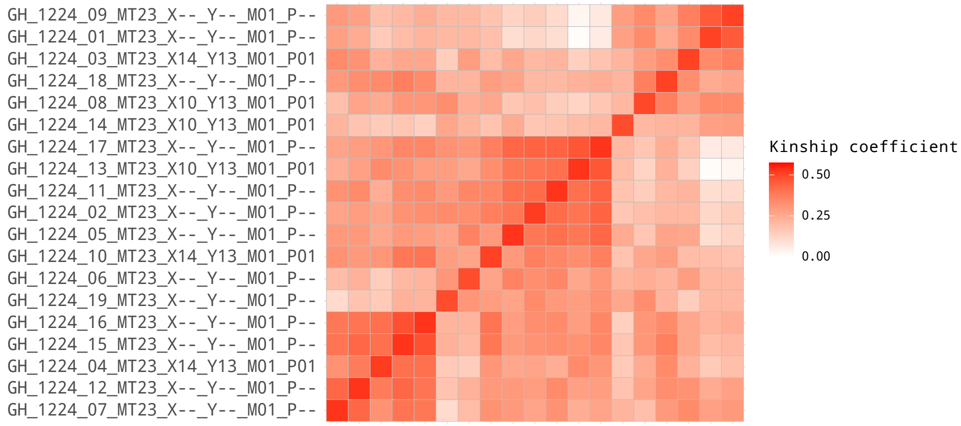
GH_1224_09_MT23_X--_Y--_M01_P--
GH_1224_01_MT23_X--_Y--_M01_P--
GH_1224_03_MT23_X14_Y13_M01_P01
GH_1224_18_MT23_X--_Y--_M01_P--
GH_1224_08_MT23_X10_Y13_M01_P01
GH_1224_14_MT23_X10_Y13_M01_P01
GH_1224_17_MT23_X--_Y--_M01_P--
GH_1224_13_MT23_X10_Y13_M01_P01
GH_1224_11_MT23_X--_Y--_M01_P--
GH_1224_02_MT23_X--_Y--_M01_P--
GH_1224_05_MT23_X--_Y--_M01_P--
GH_1224_10_MT23_X14_Y13_M01_P01
GH_1224_06_MT23_X--_Y--_M01_P--
GH_1224_19_MT23_X--_Y--_M01_P--
GH_1224_16_MT23_X--_Y--_M01_P--
GH_1224_15_MT23_X--_Y--_M01_P--
GH_1224_04_MT23_X14_Y13_M01_P01
GH_1224_12_MT23_X--_Y--_M01_P--
GH_1224_07_MT23_X--_Y--_M01_P--
Kinship coefficient
0.50
0.25
0.00

I

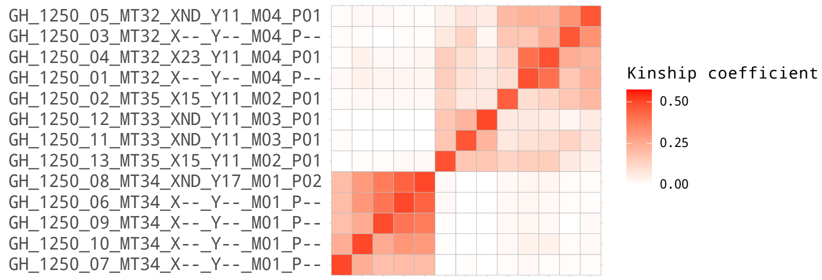
GH_1250_05_MT32_XND_Y11_M04_P01
GH_1250_03_MT32_X--_Y--_M04_P--
GH_1250_04_MT32_X23_Y11_M04_P01
GH_1250_01_MT32_X--_Y--_M04_P--
GH_1250_02_MT35_X15_Y11_M02_P01
GH_1250_12_MT33_XND_Y11_M03_P01
GH_1250_11_MT33_XND_Y11_M03_P01
GH_1250_13_MT35_X15_Y11_M02_P01
GH_1250_08_MT34_XND_Y17_M01_P02
GH_1250_06_MT34_X--_Y--_M01_P--
GH_1250_09_MT34_X--_Y--_M01_P--
GH_1250_10_MT34_X--_Y--_M01_P--
GH_1250_07_MT34_X--_Y--_M01_P--
Kinship coefficient
0.50
0.25
0.00

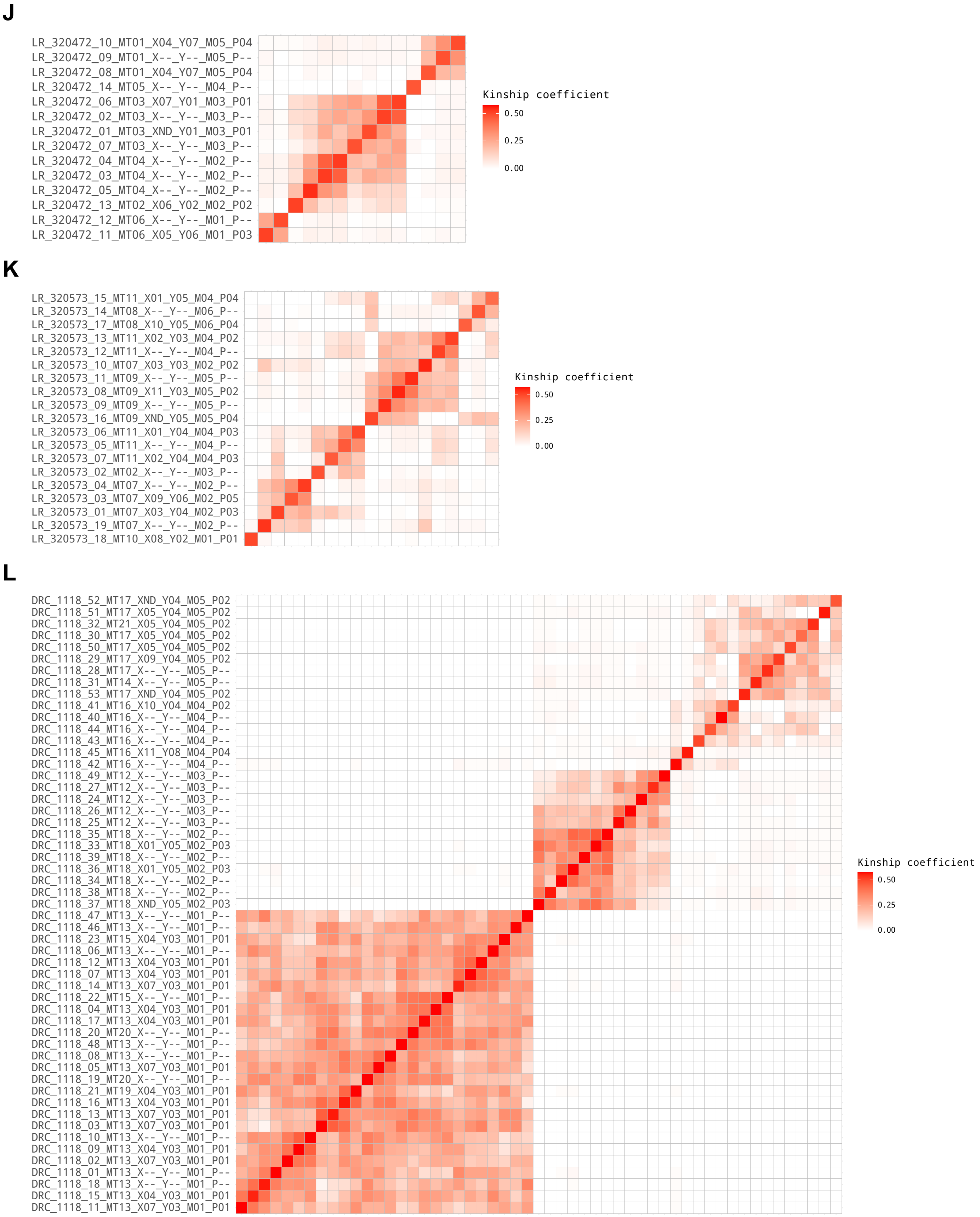
J
LR_320472_10_MT01_X04_Y07_M05_P04
LR_320472_09_MT01_X--_Y--_M05_P--
LR_320472_08_MT01_X04_Y07_M05_P04
LR_320472_14_MT05_X--_Y--_M04_P--
LR_320472_06_MT03_X07_Y01_M03_P01
LR_320472_02_MT03_X--_Y--_M03_P--
LR_320472_01_MT03_XND_Y01_M03_P01
LR_320472_07_MT03_X--_Y--_M03_P--
LR_320472_04_MT04_X--_Y--_M02_P--
LR_320472_03_MT04_X--_Y--_M02_P--
LR_320472_05_MT04_X--_Y--_M02_P--
LR_320472_13_MT02_X06_Y02_M02_P02
LR_320472_12_MT06_X--_Y--_M01_P--
LR_320472_11_MT06_X05_Y06_M01_P03
Kinship coefficient
0.50
0.25
0.00
K
LR_320573_15_MT11_X01_Y05_M04_P04
LR_320573_14_MT08_X--_Y--_M06_P--
LR_320573_17_MT08_X10_Y05_M06_P04
LR_320573_13_MT11_X02_Y03_M04_P02
LR_320573_12_MT11_X--_Y--_M04_P--
LR_320573_10_MT07_X03_Y03_M02_P02
LR_320573_11_MT09_X--_Y--_M05_P--
LR_320573_08_MT09_X11_Y03_M05_P02
LR_320573_09_MT09_X--_Y--_M05_P--
LR_320573_16_MT09_XND_Y05_M05_P04
LR_320573_06_MT11_X01_Y04_M04_P03
LR_320573_05_MT11_X--_Y--_M04_P--
LR_320573_07_MT11_X02_Y04_M04_P03
LR_320573_02_MT02_X--_Y--_M03_P--
LR_320573_04_MT07_X--_Y--_M02_P--
LR_320573_03_MT07_X09_Y06_M02_P05
LR_320573_01_MT07_X03_Y04_M02_P03
LR_320573_19_MT07_X--_Y--_M02_P--
LR_320573_18_MT10_X08_Y02_M01_P01
Kinship coefficient
0.50
0.25
0.00
L
DRC_1118_52_MT17_XND_Y04_M05_P02
DRC_1118_51_MT17_X05_Y04_M05_P02
DRC_1118_32_MT21_X05_Y04_M05_P02
DRC_1118_30_MT17_X05_Y04_M05_P02
DRC_1118_50_MT17_X05_Y04_M05_P02
DRC_1118_29_MT17_X09_Y04_M05_P02
DRC_1118_28_MT17_X--_Y--_M05_P--
DRC_1118_31_MT14_X--_Y--_M05_P--
DRC_1118_53_MT17_XND_Y04_M05_P02
DRC_1118_41_MT16_X10_Y04_M04_P02
DRC_1118_40_MT16_X--_Y--_M04_P--
DRC_1118_44_MT16_X--_Y--_M04_P--
DRC_1118_43_MT16_X--_Y--_M04_P--
DRC_1118_45_MT16_X11_Y08_M04_P04
DRC_1118_42_MT16_X--_Y--_M04_P--
DRC_1118_49_MT12_X--_Y--_M03_P--
DRC_1118_27_MT12_X--_Y--_M03_P--
DRC_1118_24_MT12_X--_Y--_M03_P--
DRC_1118_26_MT12_X--_Y--_M03_P--
DRC_1118_25_MT12_X--_Y--_M03_P--
DRC_1118_35_MT18_X--_Y--_M02_P--
DRC_1118_33_MT18_X01_Y05_M02_P03
DRC_1118_39_MT18_X--_Y--_M02_P--
DRC_1118_36_MT18_X01_Y05_M02_P03
DRC_1118_34_MT18_X--_Y--_M02_P--
DRC_1118_38_MT18_X--_Y--_M02_P--
DRC_1118_37_MT18_XND_Y05_M02_P03
DRC_1118_47_MT13_X--_Y--_M01_P--
DRC_1118_46_MT13_X--_Y--_M01_P--
DRC_1118_23_MT15_X04_Y03_M01_P01
DRC_1118_06_MT13_X--_Y--_M01_P--
DRC_1118_12_MT13_X04_Y03_M01_P01
DRC_1118_07_MT13_X04_Y03_M01_P01
DRC_1118_14_MT13_X07_Y03_M01_P01
DRC_1118_22_MT15_X--_Y--_M01_P--
DRC_1118_04_MT13_X04_Y03_M01_P01
DRC_1118_17_MT13_X04_Y03_M01_P01
DRC_1118_20_MT20_X--_Y--_M01_P--
DRC_1118_48_MT13_X--_Y--_M01_P--
DRC_1118_08_MT13_X--_Y--_M01_P--
DRC_1118_05_MT13_X07_Y03_M01_P01
DRC_1118_19_MT20_X--_Y--_M01_P--
DRC_1118_21_MT19_X04_Y03_M01_P01
DRC_1118_16_MT13_X04_Y03_M01_P01
DRC_1118_13_MT13_X07_Y03_M01_P01
DRC_1118_03_MT13_X07_Y03_M01_P01
DRC_1118_10_MT13_X--_Y--_M01_P--
DRC_1118_09_MT13_X04_Y03_M01_P01
DRC_1118_02_MT13_X07_Y03_M01_P01
DRC_1118_01_MT13_X--_Y--_M01_P--
DRC_1118_18_MT13_X--_Y--_M01_P--
DRC_1118_15_MT13_X04_Y03_M01_P01
DRC_1118_11_MT13_X07_Y03_M01_P01
Kinship coefficient
0.50
0.25
0.00

M

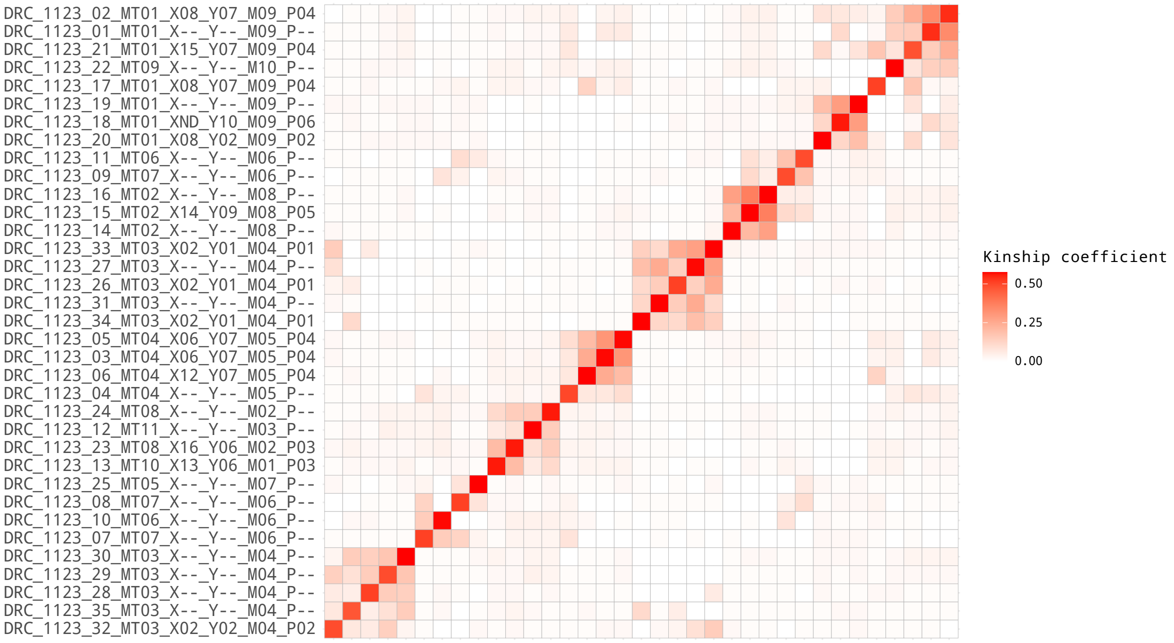

S4 Fig. Average linkage clustering of microfilariae based on kinship coefficients estimated using autosomal SNPs. Sample labels consist of "country code", "participant ID", "mf ID", "mitochondrial haplotype ID", "X-linked haplotype ID", "Y-linked haplotype ID", "maternal sibling family ID", and "paternal sibling family ID" connected by underscores. ND: not determined. (A) GH_1118, (B) GH_1123, (C) GH_1161, (D) GH_1171, (E) GH_1174, (F) GH_1177, (G) GH_1219, (H) GH_1224, (I) GH_1250, (J) LR_320472, (K) LR_320573, (L) DRC_1118, and (M) DRC_1123.
