# Supplementary material for: Genetic structuring and estimation of reproductive adults in Onchocerca volvulus: A genome-wide analysis across hosts and regions
Source: PLoS Negl Trop Dis. 2025 Jul 1;19(7):e0013221. doi: 10.1371/journal.pntd.0013221 (PMC12212510; doi:10.1371/journal.pntd.0013221)
Supplement: S5 Fig — (PDF) [file pntd.0013221.s005.pdf]

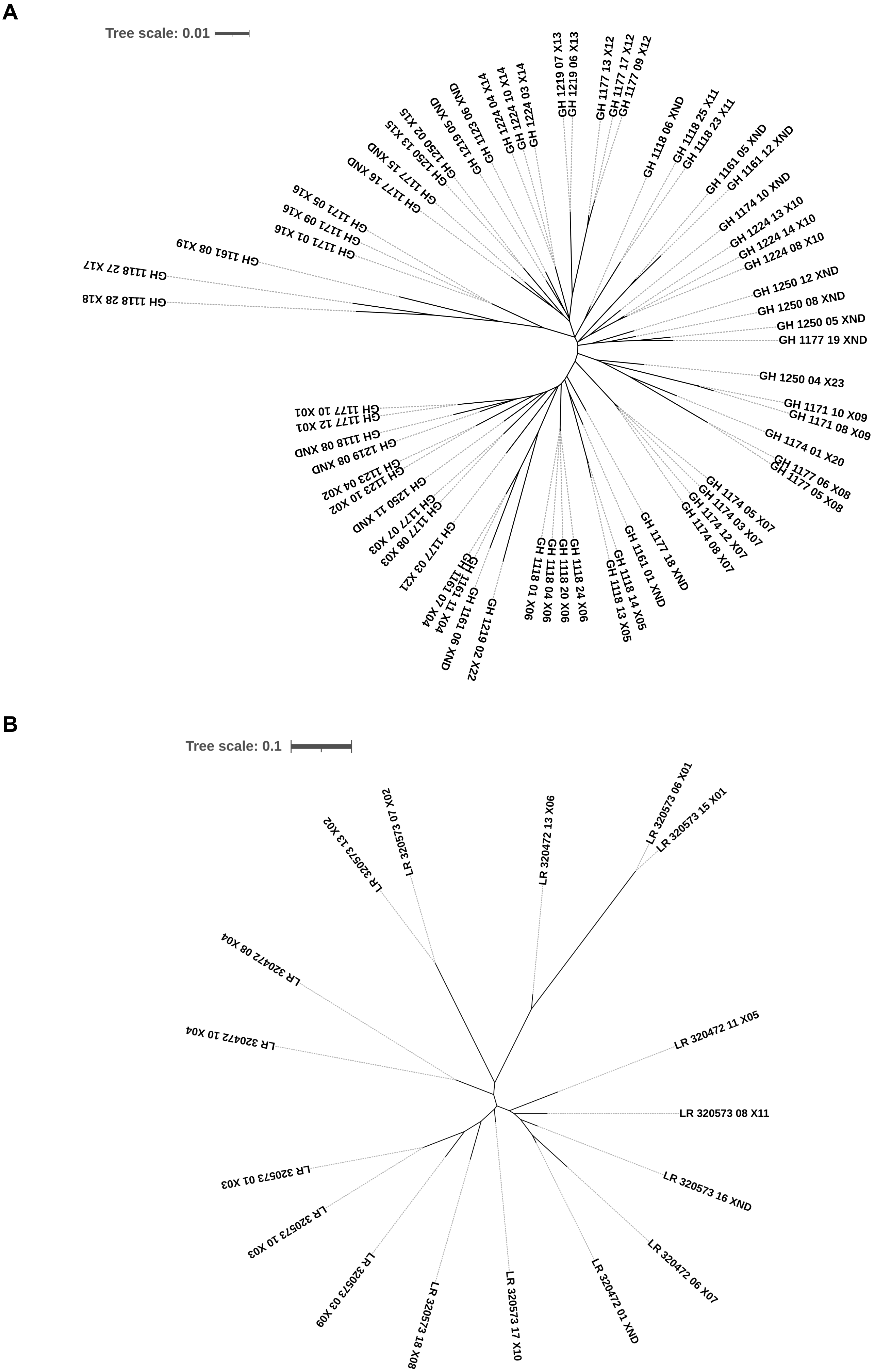
A
Tree scale: 0.01
GH 1219 07 X13
GH 1219 06 X13
GH 1177 13 X12
GH 1177 17 X12
GH 1177 09 X12
GH 1118 06 XND
GH 1118 25 X11
GH 1118 23 X11
GH 1161 05 XND
GH 1161 12 XND
GH 1174 10 XND
GH 1224 13 X10
GH 1224 14 X10
GH 1224 08 X10
GH 1250 12 XND
GH 1250 08 XND
GH 1250 05 XND
GH 1177 19 XND
GH 1250 04 X23
GH 1171 10 X09
GH 1171 08 X09
GH 1174 01 X20
GH 1177 06 X08
GH 1177 05 X08
GH 1174 05 X07
GH 1174 03 X07
GH 1174 12 X07
GH 1174 08 X07
GH 1177 18 XND
GH 1161 01 XND
GH 1118 14 X05
GH 1118 13 X05
GH 1118 24 X06
GH 1118 20 X06
GH 1118 04 X06
GH 1118 01 X06
GH 1219 02 X22
GH 1161 06 XND
GH 1161 11 X04
GH 1161 07 X04
GH 1177 03 X21
GH 1177 08 X03
GH 1177 07 X03
GH 1250 11 XND
GH 1123 10 X02
GH 1123 04 X02
GH 1219 08 XND
GH 1118 08 XND
GH 1177 12 X01
GH 1177 10 X01
GH 1118 28 X18
GH 1118 27 X17
GH 1161 08 X19
GH 1171 01 X16
GH 1171 09 X16
GH 1171 05 X16
GH 1177 16 XND
GH 1177 15 XND
GH 1250 13 X15
GH 1250 02 X15
GH 1219 05 XND
GH 1123 06 XND
GH 1224 04 X14
GH 1224 10 X14
GH 1224 03 X14
B
Tree scale: 0.1
LR 320573 13 X02
LR 320573 07 X02
LR 320472 13 X06
LR 320573 06 X01
LR 320573 15 X01
LR 320472 11 X05
LR 320573 08 X11
LR 320573 16 XND
LR 320472 06 X07
LR 320472 01 XND
LR 320573 17 X10
LR 320573 18 X08
LR 320573 03 X09
LR 320573 10 X03
LR 320573 01 X03
LR 320472 10 X04
LR 320472 08 X04

C

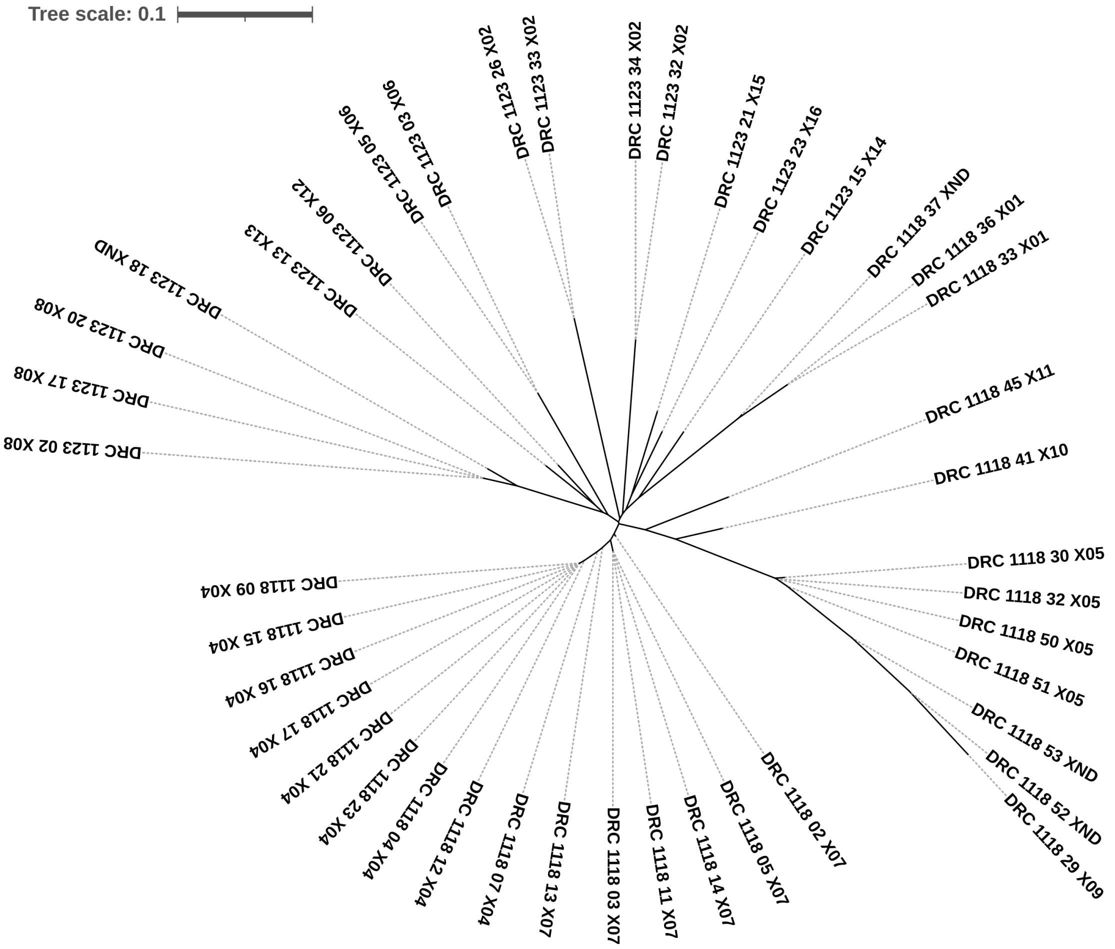

S5 Fig. Maximum likelihood phylogenetic tree of male microfilariae based on X-linked SNPs. Sample labels consist of "country code”, "participant ID”, "mf ID”, and "X-linked haplotype ID". ND: not determined. (A) Ghana. (B) Liberia. (C) DRC.
