# Supplementary material for: Genetic structuring and estimation of reproductive adults in Onchocerca volvulus: A genome-wide analysis across hosts and regions
Source: PLoS Negl Trop Dis. 2025 Jul 1;19(7):e0013221. doi: 10.1371/journal.pntd.0013221 (PMC12212510; doi:10.1371/journal.pntd.0013221)
Supplement: S6 Fig — (PDF) [file pntd.0013221.s006.pdf]

A

B

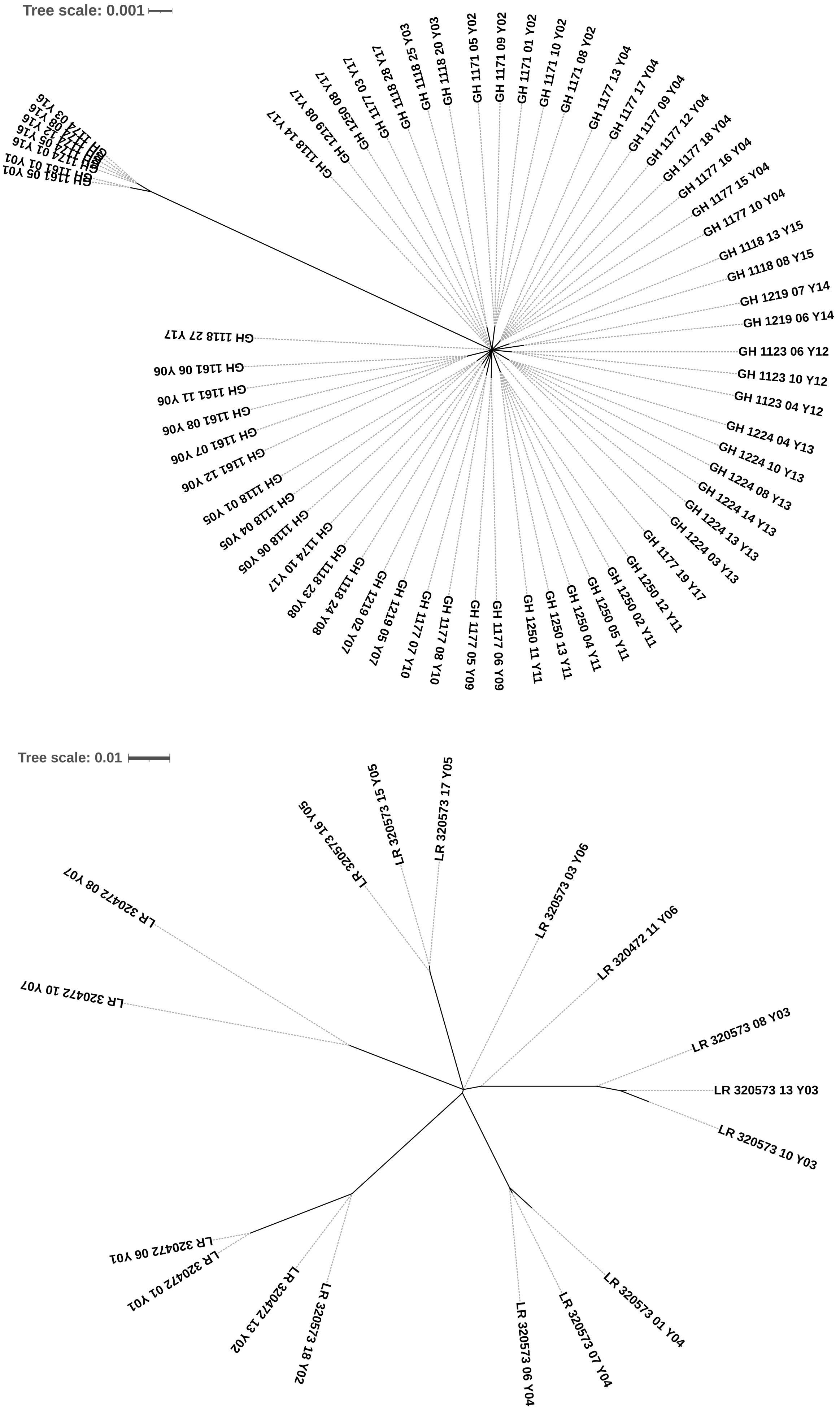
A
Tree scale: 0.001
GH 1174 03 Y16
GH 1174 08 Y16
GH 1174 12 Y16
GH 1174 05 Y16
GH 1174 01 Y16
GH 1161 01 Y01
GH 1161 05 Y01
GH 1118 14 Y17
GH 1219 08 Y17
GH 1250 08 Y17
GH 1177 03 Y17
GH 1118 28 Y17
GH 1118 25 Y03
GH 1118 20 Y03
GH 1171 05 Y02
GH 1171 09 Y02
GH 1171 01 Y02
GH 1171 10 Y02
GH 1171 08 Y02
GH 1177 13 Y04
GH 1177 17 Y04
GH 1177 09 Y04
GH 1177 12 Y04
GH 1177 18 Y04
GH 1177 16 Y04
GH 1177 15 Y04
GH 1177 10 Y04
GH 1118 13 Y15
GH 1118 08 Y15
GH 1219 07 Y14
GH 1219 06 Y14
GH 1123 06 Y12
GH 1123 10 Y12
GH 1123 04 Y12
GH 1224 04 Y13
GH 1224 10 Y13
GH 1224 08 Y13
GH 1224 14 Y13
GH 1224 13 Y13
GH 1224 03 Y13
GH 1177 19 Y17
GH 1250 12 Y11
GH 1250 02 Y11
GH 1250 05 Y11
GH 1250 04 Y11
GH 1250 13 Y11
GH 1250 11 Y11
GH 1177 06 Y09
GH 1177 05 Y09
GH 1177 08 Y10
GH 1177 07 Y10
GH 1219 05 Y07
GH 1219 02 Y07
GH 1118 24 Y08
GH 1118 23 Y08
GH 1174 10 Y17
GH 1118 06 Y05
GH 1118 04 Y05
GH 1118 01 Y05
GH 1161 12 Y06
GH 1161 07 Y06
GH 1161 08 Y06
GH 1161 11 Y06
GH 1161 06 Y06
GH 1118 27 Y17
B
Tree scale: 0.01
LR 320573 16 Y05
LR 320573 15 Y05
LR 320573 17 Y05
LR 320573 03 Y06
LR 320472 11 Y06
LR 320573 08 Y03
LR 320573 13 Y03
LR 320573 10 Y03
LR 320573 01 Y04
LR 320573 07 Y04
LR 320573 06 Y04
LR 320573 18 Y02
LR 320472 13 Y02
LR 320472 01 Y01
LR 320472 06 Y01
LR 320472 10 Y07
LR 320472 08 Y07

C

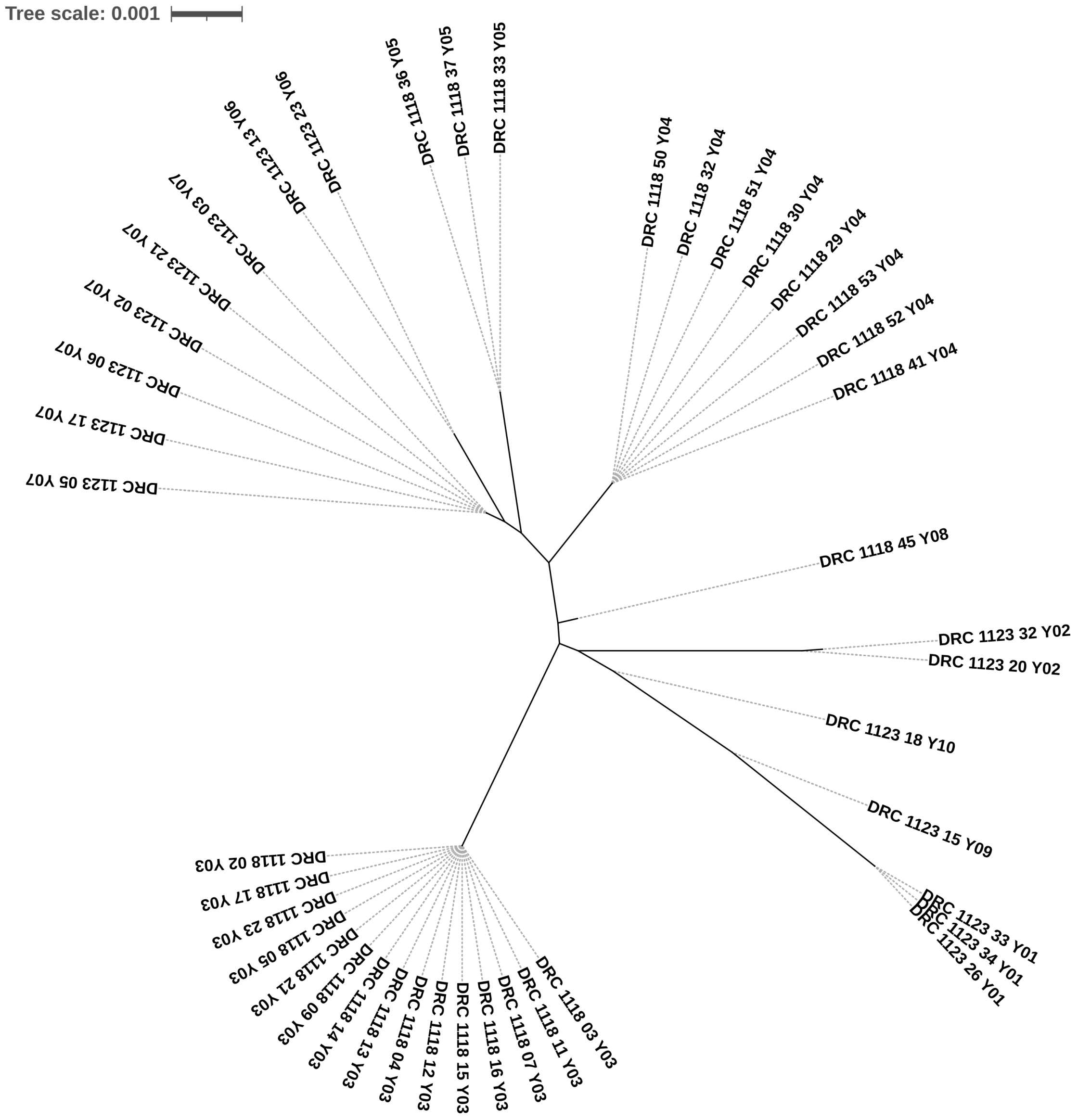

S6 Fig. Maximum likelihood phylogenetic tree of male microfilariae based on Y-linked SNPs. Sample labels consist of "country code”, "participant ID”, "mf ID”, and ”Y-linked haplotype ID". ND: not determined. (A) Ghana. (B**)** Liberia. (C) DRC.
