# Supplementary material for: Genetic structuring and estimation of reproductive adults in Onchocerca volvulus: A genome-wide analysis across hosts and regions
Source: PLoS Negl Trop Dis. 2025 Jul 1;19(7):e0013221. doi: 10.1371/journal.pntd.0013221 (PMC12212510; doi:10.1371/journal.pntd.0013221)
Supplement: S8 Fig — (PDF) [file pntd.0013221.s008.pdf]

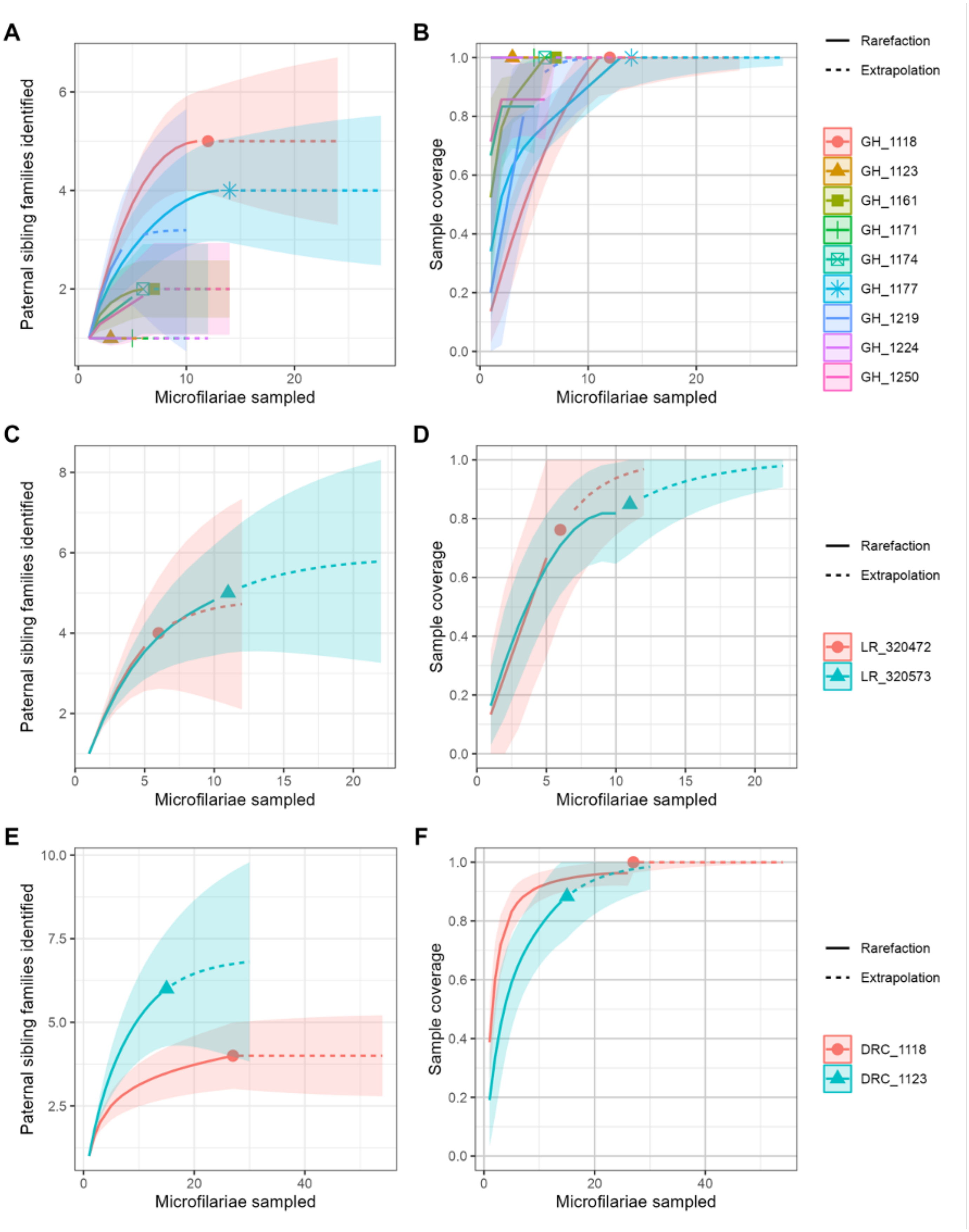

S8 Fig. Rarefaction and extrapolation curves for *Onchocerca volvulus* paternal sibling families identified from hosts using male microfilariae. Shaded areas represent 95% confidence intervals. (A-B) Ghana. (C-D) Liberia. (E-F) DRC.
