# Supplementary material for: Genetic structuring and estimation of reproductive adults in Onchocerca volvulus: A genome-wide analysis across hosts and regions
Source: PLoS Negl Trop Dis. 2025 Jul 1;19(7):e0013221. doi: 10.1371/journal.pntd.0013221 (PMC12212510; doi:10.1371/journal.pntd.0013221)
Supplement: S9 Fig — (PDF) [file pntd.0013221.s009.pdf]

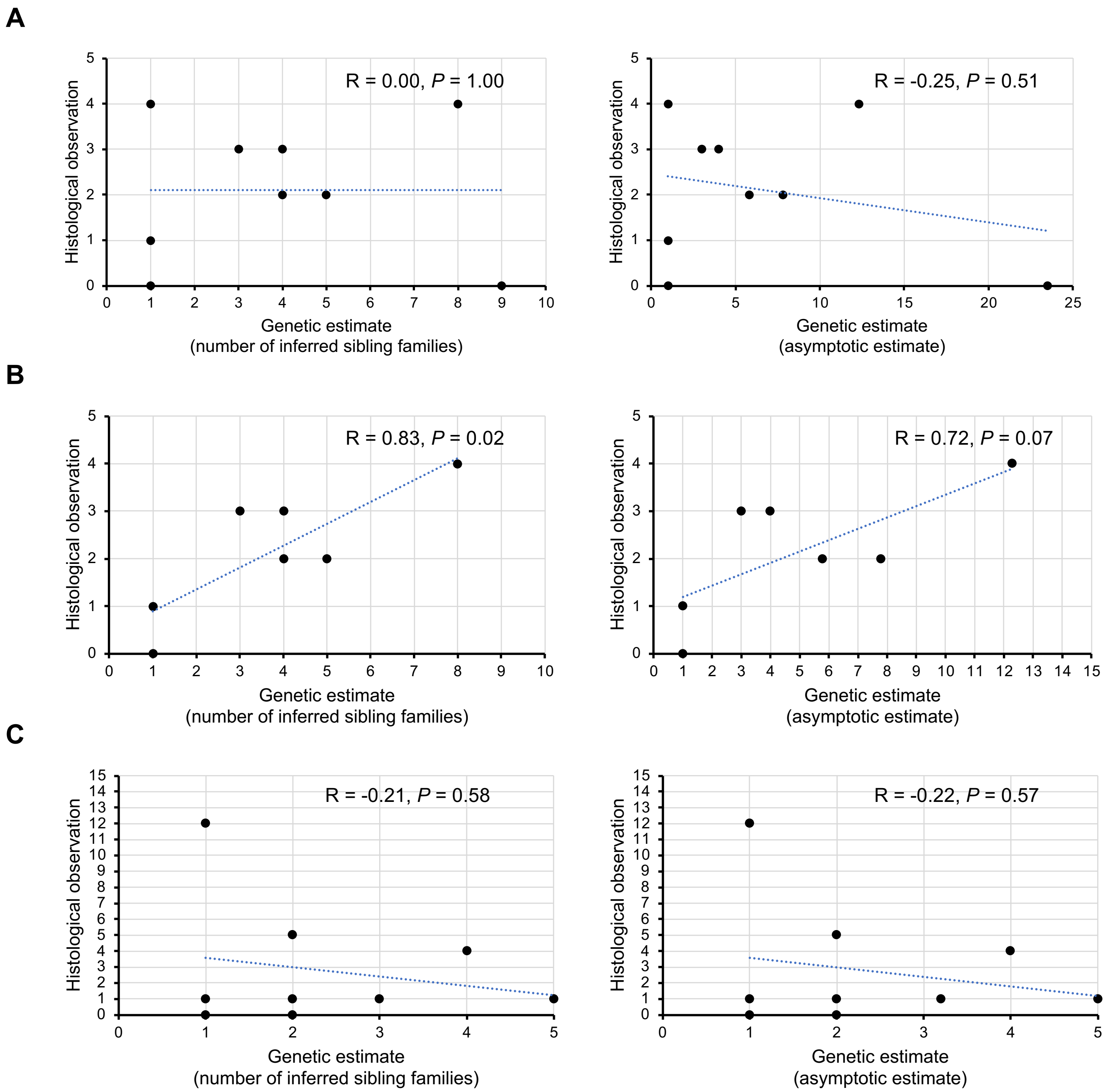

S9 Fig. Correlation between genetically estimated and histologically observed *Onchocerca volvulus* adult worm counts for participants from Ghana. (A) Adult female worm counts from 9 participants. (B) Adult female worm counts from 7 participants, excluding GH_1118 and GH_1171. (C) Adult male worm counts from 9 participants.
