# Supplementary material for: Genetic structuring and estimation of reproductive adults in Onchocerca volvulus: A genome-wide analysis across hosts and regions
Source: PLoS Negl Trop Dis. 2025 Jul 1;19(7):e0013221. doi: 10.1371/journal.pntd.0013221 (PMC12212510; doi:10.1371/journal.pntd.0013221)
Supplement: S1 Table — (PDF) [file pntd.0013221.s010.pdf]

S1 Table. Anthelmintic treatment history for each participant and microfilariae density pre- and post-treatment.

Country	Participant ID	Treatment*	Mf density (mf/mg)			Mf sample collection
			Pre-treatment	Post-treatment		
				12 months	18 months	
Ghana	GH_1010	IDA$^{\text{(3 dose)}}$	35.56	1.6	20.27	18 months post-treatment
	GH_1013	IA$^{\text{(1 dose)}}$	34.39	-	14.84	
	GH_1015	IDA$^{\text{(3 dose)}}$	12.45	8.46	21.82	
	GH_1036	IDA$^{\text{(1 dose)}}$	56.98	2.26	13.53	
	GH_1086	IA$^{\text{(1 dose)}}$	45.63	1.34	12.46	
	GH_1118	IA$^{\text{(1 dose)}}$	282.8	18.7	42.84	
	GH_1123	IA$^{\text{(1 dose)}}$	19.6	12.4	13.9	
	GH_1161	IDA$^{\text{(3 dose)}}$	73.28	8.15	57.59	
	GH_1171	IDA$^{\text{(1 dose)}}$	65.03	2.78	17.89	
	GH_1174	IA$^{\text{(1 dose)}}$	122.3	11	29.54	
	GH_1177	IDA$^{\text{(1 dose)}}$	236.9	15.7	41.8	
	GH_1182	IA$^{\text{(1 dose)}}$	158.36	10.43	6.02	
	GH_1213	IDA$^{\text{(3 dose)}}$	144.58	23.25	1.67	
	GH_1219	IA$^{\text{(1 dose)}}$	315.8	21.9	29.4	
	GH_1224	IDA$^{\text{(1 dose)}}$	116.5	6.9	42.21	
	GH_1250	IDA$^{\text{(3 dose)}}$	110.77	13.19	17.41	
Liberia	LR_320472	naïve	-	-	-	-
	LR_320562					
	LR_320573					
DRC	DRC_1118	-	141.75	-	-	Pre-treatment
	DRC_1123		86.25			
	DRC_1157		240.75			
	DRC_1231		245.5			
	DRC_1284		77.25			

*I, Ivermectin; D, Diethylcarbamazine; A, Albendazole
