# Supplementary material for: Genetic structuring and estimation of reproductive adults in Onchocerca volvulus: A genome-wide analysis across hosts and regions
Source: PLoS Negl Trop Dis. 2025 Jul 1;19(7):e0013221. doi: 10.1371/journal.pntd.0013221 (PMC12212510; doi:10.1371/journal.pntd.0013221)
Supplement: S4 Table — (PDF) [file pntd.0013221.s013.pdf]

S4 Table. Comparison of the number of adult worms estimated from microfilariae genetic data and those identified through histological analysis of nodules.

Participant ID	Estimates based on microfilariae genetic data		Histological Data					
	Adult female	Adult male	Evaluable nodule	Adult female (total)	Adult female (alive)	Adult female (normal embryogenesis)	Adult male (alive)	Nodules with microfilariae
GH_1118	9	5	1	1	1	0	1	0
GH_1123	1	1	2	3	2	0	0	0
GH_1161	5	2	2	7	6	2	1	1
GH_1171	1	1	12	23	11	4	12	2
GH_1174	4	2	4	8	5	2	0	0
GH_1177	8	4	5	15	7	4	4	3
GH_1219	3	3	5	13	10	3	1	3
GH_1224	1	1	1	1	1	1	1	0
GH_1250	4	2	6	9	7	3	5	2
