# Supplementary material for: Genetic structuring and estimation of reproductive adults in Onchocerca volvulus: A genome-wide analysis across hosts and regions
Source: PLoS Negl Trop Dis. 2025 Jul 1;19(7):e0013221. doi: 10.1371/journal.pntd.0013221 (PMC12212510; doi:10.1371/journal.pntd.0013221)
Supplement: S1 Text — (PDF) [file pntd.0013221.s014.pdf]

S1 Text. Bioinformatics pipeline and command-line arguments used in the analysis

```
## Adapter and quality trimming of paired-end reads using Trimmomatic v0.39

java -jar trimmomatic-0.39.jar PE -threads [cpu#] [R1.fastq.gz] [R2.fastq.gz] [forward_paired.fastq.gz]
[forward_unpaired.fastq.gz] [reverse_paired.fastq.gz] [reverse_unpaired.fastq.gz]
ILLUMINACLIP:[adapters.fasta]:2:30:10:2 LEADING:3 TRAILING:3 SLIDINGWINDOW:4:15 MINLEN:36


## Reference genome alignment using BWA v0.7.17

bwa index [reference_genome.fasta]

(seqtk mergepe [forward_paired.fastq.gz] [reverse_paired.fastq.gz]; zcat [forward_unpaired.fastq.gz]
[reverse_unpaired.fastq.gz]) | bwa mem -M -t [cpu#] -p -o [alignment.sam] [reference_genome.fasta] -


## Converting SAM to BAM and adding read group information using Picard v2.27.5

java -jar picard.jar AddOrReplaceReadGroups -I [alignment.sam] -O [alignment.bam] --RGID [RGID] --RGLB [RGLB] --
RGPL ILLUMINA --RGSM [RGSM] --RGPU [RGPU] --SORT_ORDER coordinate --CREATE_INDEX true


## Merging BAM files by sample using Samtools v1.16

samtools merge -f -o [sample.bam] -b [bam.list]

samtools index [sample.bam]


## Removing PCR and optical duplicates using Picard v2.27.5

java -jar picard.jar MarkDuplicates -I [sample.bam] -O [sample_dedup.bam] --REMOVE_DUPLICATES true --
METRICS_FILE [sample.log] --ASSUME_SORTED true --VALIDATION_STRINGENCY LENIENT --CREATE_INDEX true


## Calculating genome coverage using Samtools v1.16

samtools stats [sample_dedup.bam] --cov-threshold 10 -t [target-region]


## Nuclear DNA variant calling and filtering using GATK v4.3.0.0

java -jar gatk.jar HaplotypeCaller -I [sample_dedup.bam] -R [reference_genome.fasta] -O [sample.g.vcf.gz] --min-
base-quality-score 20 --minimum-mapping-quality 30 -ERC GVCF

java -jar gatk.jar CombineGVCFs -R [reference_genome.fasta] -V [gvcf.list] -O [combined.g.vcf.gz]

java -jar gatk.jar GenotypeGVCFs -R [reference_genome.fasta] -V [combined.g.vcf.gz] -O [output.vcf.gz]

java -jar gatk.jar SelectVariants -R [reference_genome.fasta] -V [output.vcf.gz] -select-type SNP -O
[SNP.vcf.gz]

java -jar gatk.jar VariantFiltration -R [reference_genome.fasta] -V [SNP.vcf.gz] -O [SNP.filtered.vcf.gz] --
filter-name "QD2" -filter "QD < 2.0" --filter-name "QUAL30" -filter "QUAL < 30.0" --filter-name "FS60" -filter
"FS > 60.0" --filter-name "MQ40" -filter "MQ < 40.0" --filter-name "SOR3" -filter "SOR > 3.0" --filter-name
"MQRankSum-10" -filter "MQRankSum < -10.0" --filter-name "ReadPosRankSum-10" -filter "ReadPosRankSum < -10.0" --
filter-name "ReadPosRankSum10" -filter "ReadPosRankSum > 10.0" --filter-name "DP" -filter "DP >
[median_depth*2]"

zgrep -E '^#|PASS' [SNP.filtered.vcf.gz] > [SNP.filtered.passed.vcf]
```

```
vcftools --vcf [SNP.filtered.passed.vcf] --bed [target-region.bed] --depth --out [output_prefix]

vcftools --vcf [SNP.filtered.passed.vcf] --bed [target-region.bed] --missing-indv --out [output_prefix]

vcftools --vcf [SNP.filtered.passed.vcf] --bed [target-region.bed] --het --out [output_prefix]
```

Mitochondrial DNA variant calling using GATK v4.3.0.0

```
java -jar gatk.jar HaplotypeCaller -I [sample_dedup.bam] -R [reference_genome.fasta] --linked-de-bruijn-graph true -L [mitochondria.bed] --sample-ploidy 1 -O [sample.ploidy_1.g.vcf.gz] --minimum-mapping-quality 30 -ERC GVCF

java -jar gatk.jar CombineGVCFs -R [reference_genome.fasta] -V [gvcf.list] -O [combined.ploidy_1.g.vcf.gz]

java -jar gatk.jar GenotypeGVCFs -R [reference_genome.fasta] -V [combined.ploidy_1.g.vcf.gz] -O [output.ploidy_1.vcf.gz]

java -jar gatk.jar SelectVariants -R [reference_genome.fasta] -V [output.ploidy_1.vcf.gz] -select-type SNP -O [SNP.ploidy_1.vcf.gz]

java -jar gatk.jar VariantFiltration -R [reference_genome.fasta] -V [SNP.ploidy_1.vcf.gz] -O [SNP.filtered.ploidy_1.vcf.gz] --filter-name "QD2" -filter "QD < 2.0" --filter-name "QUAL30" -filter "QUAL < 30.0" --filter-name "FS60" -filter "FS > 60.0" --filter-name "MQ40" -filter "MQ < 40.0" --filter-name "SOR3" -filter "SOR > 3.0" --filter-name "MQRankSum-10" -filter "MQRankSum < -10.0" --filter-name "ReadPosRankSum-10" -filter "ReadPosRankSum < -10.0" --filter-name "ReadPosRankSum10" -filter "ReadPosRankSum > 10.0" --filter-name "DP20" -filter "DP < 20"

zgrep -E '^#|PASS' [SNP.filtered.ploidy_1.vcf.gz] > [SNP.filtered.passed.ploidy_1.vcf]
```

Identifying and excluding nuclear mitochondrial DNA (NUMT) positions

```
java -jar gatk.jar HaplotypeCaller -I [sample_dedup.bam] -R [reference_genome.fasta] --linked-de-bruijn-graph true -L [mitochondria.bed] --sample-ploidy 2 -O [sample.ploidy_2.g.vcf.gz] --minimum-mapping-quality 30 -ERC GVCF

java -jar gatk.jar CombineGVCFs -R [reference_genome.fasta] -V [gvcf.list] -O [combined.ploidy_2.g.vcf.gz]

java -jar gatk.jar GenotypeGVCFs -R [reference_genome.fasta] -V [combined.ploidy_2.g.vcf.gz] -O [output.ploidy_2.vcf.gz]

java -jar gatk.jar SelectVariants -R [reference_genome.fasta] -V [output.ploidy_2.vcf.gz] -select-type SNP -O [SNP.ploidy_2.vcf.gz]

java -jar gatk.jar VariantFiltration -R [reference_genome.fasta] -V [SNP.ploidy_2.vcf.gz] -O [SNP.filtered.ploidy_2.vcf.gz] --filter-name "QD2" -filter "QD < 2.0" --filter-name "QUAL30" -filter "QUAL < 30.0" --filter-name "FS60" -filter "FS > 60.0" --filter-name "MQ40" -filter "MQ < 40.0" --filter-name "SOR3" -filter "SOR > 3.0" --filter-name "MQRankSum-10" -filter "MQRankSum < -10.0" --filter-name "ReadPosRankSum-10" -filter "ReadPosRankSum < -10.0" --filter-name "ReadPosRankSum10" -filter "ReadPosRankSum > 10.0" --filter-name "DP20" -filter "DP < 20"

zgrep -E '^#|PASS' [SNP.filtered.ploidy_2.vcf.gz] > [SNP.filtered.passed.ploidy_2.vcf]

bcftools query -f '%CHROM\t%POS\t%REF\t%ALT[\t%GT]\n' [SNP.filtered.passed.ploidy_2.vcf] | awk 'BEGIN{OFS="\t"}{het=0; for(i=5;i<=NF;i++){if($i=="0/1" || $i=="1/0"){het=1; break;}} if(het) print $1,$2,$3,$4;}' > [non-monoallelic.positions]

vcftools --vcf [SNP.filtered.passed.ploidy_1.vcf] --exclude-positions [non-monoallelic.positions] --recode --recode-INFO-all --out [SNP.filtered.passed.non-monoallelic_removed.ploidy_1.vcf]
```

Filtering mitochondrial singleton SNPs using VCFtools v0.1.16

```
vcftools --vcf [SNP.filtered.passed.non-monoallelic_removed.ploidy_1.vcf] --singletons --out [output_prefix]
```

```
vcftools --vcf [SNP.filtered.passed.non-monoallelic_removed.ploidy_1.vcf] --exclude-positions [output_prefix.singletons] --recode --recode-INFO-all --out [SNP.filtered.passed.non-monoallelic_removed.singleton_removed.ploidy_1.vcf]


## Converting VCF to TAB format using VCFtools v0.1.16

cat [SNP.filtered.passed.non-monoallelic_removed.singleton_removed.ploidy_1.vcf] | /vcftools-0.1.16/src/perl/vcf-to-tab | grep -v '*' | sed 's|\./|N/|g' > [mito.tab]


## Converting TAB to FASTA format (https://code.google.com/archive/p/vcf-tab-to-fasta/)

perl vcf_tab_to_fasta_alignment.pl -i [mito.tab] > [mito.fasta]


## Converting FASTA to NEXUS format using trimAl v1.4.1

trimal -in [mito.fasta] -out [mito.nex] -nexus


## Discriminant analysis of principal components (DAPC)

plink --vcf [SNP.filtered.passed.vcf.gz] --double-id --allow-extra-chr --set-missing-var-ids @:# --maf 0.05 --indep-pairwise 200 5 0.2 --out [prune]

plink --vcf [SNP.filtered.passed.vcf.gz] --double-id --allow-extra-chr --set-missing-var-ids @:# --chr OM1, OM3, OM4 --extract [prune.in] --geno 0.01 --recode vcf --out [autosome]


## DAPC using adegenet v2.1.10 in R

library(adegenet)

library(vcfR)

vcf <- read.vcfR("autosome.vcf")

pop <- read.table("group_membership.txt", header=FALSE, sep="\t", stringsAsFactors = TRUE)

gl1 <- vcfR2genlight(vcf)

gl1@pop <- pop$V1

gl2 <- tab(gl1, NA.method="mean")

xval = xvalDapc(gl2, grp=as.factor(unlist(gl1$pop)), training.set=0.9,  result="groupMean", center=TRUE, scale=FALSE, n.rep=500, n.pca=NULL, parallel="snow")

mydapc <- dapc(gl1, pop=as.factor(unlist(gl1$pop)), var.loadings=TRUE, pca.info=TRUE)

coordinates <- as.data.frame(mydapc$ind.coord)

assignment_rate <- summary(mydapc)$assign.per.pop*100


## Sibship reconstruction and parentage inference

plink --vcf [SNP.filtered.passed.vcf.gz] --double-id --allow-extra-chr --set-missing-var-ids @:# --chr OM1, OM3, OM4 --mac 4 --geno 0.05 --make-bed --out [output_prefix]


## Estimating kinship coefficients using GENESIS v2.34 in R

library(GENESIS)

library(SNPRelate)
```

```
library(GWASTools)

snpgdsBED2GDS(bed.fn = "output_prefix.bed", bim.fn = "output_prefix.bim", fam.fn = "output_prefix.fam",
out.gdsfn = "output_prefix.gds")

genoData <- GenotypeData(GdsGenotypeReader(filename = "output_prefix.gds"))

gds <- snpgdsOpen("output_prefix.gds", allow.duplicate=TRUE)

snpset <- snpgdsLDpruning(gds, method="corr", slide.max.bp=10e6, ld.threshold=sqrt(0.2), verbose=FALSE)

pruned <- unlist(snpset, use.names=FALSE)

king <- snpgdsIBDKING(gds, snp.id=NULL, verbose=FALSE)

kingMat <- king$kinship

mypcair <- pcair(gds, kinobj=kingMat, kin.thresh=2^(-7/2), divobj=kingMat, div.thresh=-2^(-7/2),
snp.include=pruned)

iterator <- GenotypeBlockIterator(genoData)

mypcrelate <- pcrelate(iterator, pcs = mypcair$vectors[,1:4], ibd.probs = TRUE, training.set = mypcair$unrels,
BPPARAM = BiocParallel::SerialParam())

mymatrix <- as.matrix(pcrelateToMatrix(mypcrelate, scaleKin = 1))


## Clustering kinship matrix using ggcorrplot v0.1.4.1

library(ggcorrplot)

ggcorrplot(mymatrix, hc.order = T, hc.method = "average")


## X-linked haplotype analysis

## Filtering variants with BCFtools v1.9

bcftools view --samples-file [male_sample.list] --targets-file [X-chr_non_PAR.bed] -f "PASS"
[SNP.filtered.passed.vcf.gz] | bcftools view -o [X-chr_male.vcf.gz] -g ^het --min-ac 4:minor -

bcftools index -t [X-chr_male.vcf.gz]


## Removing missing genotype loci using PLINK v1.9

plink --vcf [X-chr_male.vcf] --double-id --allow-extra-chr --set-missing-var-ids @:# --geno 0 --recode vcf --out
[X-chr_male.no_missing]


## Converting VCF to PHYLIP format using vcf2phylip v2.8

python3 vcf2phylip.py --input [X-chr_male.no_missing.vcf] --output-folder [output_folder]


## Building phylogenetic trees using IQ-TREE v1.6.12

iqtree -s [X-chr_male.no_missing.phy]


## Clustering sequences using TreeCluster v1.0.4

TreeCluster.py -i [X-chr_male.no_missing.phy.treefile] -o [output] -t [threshold] -m max


## Rarefaction analysis using iNEXT v3.0.0 in R
```

```
## Data from two participants from Liberia are used as examples: LR_320472 (mf count = 14; 5 maternal families) and LR_320573 (mf count = 19; 6 maternal families).

library(iNEXT)

library(ggplot2)

data <- list("LR_320472" = c(2, 4, 4, 1, 3), "LR_320573" = c(1, 5, 1, 6, 4, 2))

out <- iNEXT(data, q = 0, datatype = "abundance", knots = 40, se = TRUE, conf = 0.95, nboot = 999)

df <- fortify(out, type = 1)

point <- df[df$Method == "Observed", ]

line <- df[df$Method != "Observed", ]

ggplot(df, aes(x = x, y = y, colour = Assemblage)) + geom_point(data = point) + geom_line(data = line, aes(linetype = Method)) + geom_ribbon(aes(ymin = y.lwr, ymax = y.upr, fill = Assemblage), alpha = 0.2) + labs(x = "Microfilariae sampled", y = "Maternal sibling families identified")

df_SC <- fortify(out, type = 2)

point_SC <- df_SC[df_SC$Method == "Observed", ]

line_SC <- df_SC[df_SC$Method != "Observed", ]

ggplot(df_SC, aes(x = x, y = y, colour = Assemblage)) + geom_point(data = point_SC) + geom_line(data = line_SC, aes(linetype = Method)) + geom_ribbon(aes(ymin = y.lwr, ymax = y.upr, fill = Assemblage), alpha = 0.2) + labs(x = "Microfilariae sampled", y = "Sample coverage")


## Linkage disequilibrium decay analysis using PopLDdecay v3.42

java -jar gatk.jar SelectVariants -L [autosome.bed] -V [SNP.filtered.passed.vcf.gz] -O [SNP.filtered.passed.autosome.vcf.gz]

plink2 --vcf [SNP.filtered.passed.autosome.vcf.gz] --double-id --allow-extra-chr --set-missing-var-ids @:# --keep [ghana_female_sample.list] --out [output_prefix] --king-cutoff 0.08838835 --geno 0 --mac 4

PopLDdecay -InVCF [chromosome.vcf.gz] -SubPop [unrelated_female.list] -MAF 0.05 -OutStat [output_prefix]


## Preparing VCFs containing both invariant (i.e., monomorphic) and variant sites using GATK v4.3.0.0

java -jar gatk.jar GenotypeGVCFs -R [reference_genome.fasta] -V [combined.g.vcf.gz] -all-sites -L [chromosome] -O [chromosome_allsites.vcf.gz]

java -jar gatk.jar SelectVariants -R [reference_genome.fasta] -V [chromosome_allsites.vcf.gz] --select-type-to-exclude INDEL -O [chromosome_allsites.snp.vcf.gz]

java -jar gatk.jar VariantFiltration -R [reference_genome.fasta] -V [chromosome_allsites.snp.vcf.gz] -O [chromosome_allsites.snp.filtered.vcf.gz] --filter-name "QD2" -filter "QD < 2.0" --filter-name "QUAL30" -filter "QUAL < 30.0" --filter-name "FS60" -filter "FS > 60.0" --filter-name "MQ40" -filter "MQ < 40.0" --filter-name "SOR3" -filter "SOR > 3.0" --filter-name "MQRankSum-10" -filter "MQRankSum < -10.0" --filter-name "ReadPosRankSum-10" -filter "ReadPosRankSum < -10.0" --filter-name "ReadPosRankSum10" -filter "ReadPosRankSum > 10.0" --filter-name "DP" -filter "DP > [median_depth*2]"

vcftools --gzvcf [chromosome_allsites.snp.filtered.vcf.gz] --max-missing 0.8 --max-maf 0 --recode --out [chromosome.snp.filtered.invariant.vcf]

vcftools --gzvcf [chromosome_allsites.snp.filtered.vcf.gz] --max-missing 0.8 --mac 1 --recode --out [chromosome.snp.filtered.variant.vcf]

grep -E '^#|PASS' [chromosome.snp.filtered.variant.vcf] > [chromosome.snp.filtered.variant.passed.vcf]

bcftools concat --allow-overlaps [chromosome.snp.filtered.invariant.vcf] [chromosome.snp.filtered.variant.passed.vcf] -O z -o [chromosome.concat.vcf.gz]
```

```
## Estimating nucleotide diversity using Pixy v1.2.7

pixy --stats pi fst dxy --vcf [chromosome.concat.vcf.gz] --populations [unrelated_female.list] --window_size
10000 --n_cores [cpu#] --chromosomes [chromosome] --output_prefix [output_prefix]


## Estimating Tajima's D using VCFtools v0.1.16

vcftools --gzvcf [chromosome.vcf.gz] --TajimaD [window size] --keep [unrelated_female.list] --out
[output_prefix]


## Analyzing haplotype blocks using PLINK v1.9

plink --vcf [SNP.filtered.passed.vcf.gz] --double-id --allow-extra-chr --set-missing-var-ids @:# --chr
[chromosome] --keep [unrelated_female.list] --blocks no-pheno-req --blocks-max-kb 1000 --out [output_prefix]
```
